# The association between SAα2,3Gal occurrence frequency and avian influenza viral load in mallards (*Anas platyrhynchos*) and blue-winged teals (*Spatula discors*)

**DOI:** 10.1186/s12917-020-02642-7

**Published:** 2020-11-10

**Authors:** Amanda C. Dolinski, Mark D. Jankowski, Jeanne M. Fair, Jennifer C. Owen

**Affiliations:** 1 Department of Fisheries and Wildlife, Michigan State University, East Lansing, MI USA; 2 U.S. Environmental Protection Agency, Seattle, WA USA; 3 Los Alamos National Laboratory, Biosecurity & Public Health, Los Alamos, NM USA; 4 Department of Large Animal Clinical Sciences, Michigan State University, East Lansing, MI USA

**Keywords:** Avian, Influenza, Lectin histochemistry, Super-shedder

## Abstract

**Background:**

Individual heterogeneity in pathogen load can affect disease transmission dynamics; therefore, identifying intrinsic factors responsible for variation in pathogen load is necessary for determining which individuals are prone to be most infectious. Because low pathogenic avian influenza viruses (LPAIV) preferentially bind to alpha-2,3 sialic acid receptors (SAα2,3Gal) in the intestines and bursa of Fabricius in wild ducks (*Anas* and *Spatula* spp.), we investigated juvenile mallards (*Anas platyrhyncos*) and blue-winged teals (*Anas discors*) orally inoculated with A/northern pintail/California/44221–761/2006 (H5N9) and the virus titer relationship to occurrence frequency of SAα2,3Gal in the intestines and bursa. To test the natural variation of free-ranging duck populations, birds were hatched and raised in captivity from eggs collected from nests of free-ranging birds in North Dakota, USA. Data generated from qPCR were used to quantify virus titers in cloacal swabs, ileum tissue, and bursa of Fabricius tissue, and lectin histochemistry was used to quantify the occurrence frequency of SAα2,3Gal. Linear mixed models were used to analyze infection status, species, and sex-based differences. Multiple linear regression was used to analyze the relationship between virus titer and SAα2,3Gal occurrence frequency.

**Results:**

In mallards, we found high individual variation in virus titers significantly related to high variation of SAα2,3Gal in the ileum. In contrast to mallards, individual variation in teals was minimal and significant relationships between virus titers and SAα2,3Gal were not determined. Collectively, teals had both higher virus titers and a higher occurrence frequency of SAα2,3Gal compared to mallards, which may indicate a positive association between viral load and SAα2,3Gal. Statistically significant differences were observed between infected and control birds indicating that LPAIV infection may influence the occurrence frequency of SAα2,3Gal, or vice versa, but only in specific tissues.

**Conclusions:**

The results of this study provide quantitative evidence that SAα2,3Gal abundance is related to LPAIV titers; thus, SAα2,3Gal should be considered a potential intrinsic factor influencing variation in LPAIV load.

**Supplementary Information:**

The online version contains supplementary material available at 10.1186/s12917-020-02642-7.

## Background

Wild waterfowl are the natural reservoir for avian influenza viruses (AIV) and a source of infection for domestic poultry [1–3]. Highly pathogenic avian influenza virus (HPAIV), which causes devastating impacts to poultry worldwide with some strains fatal to humans, originates from strains of low pathogenic avian influenza virus (LPAIV) circulating in wild ducks [4]. LPAIV is transmitted most efficiently via the fecal-oral route [5] and transmitted to poultry via direct contact, contaminated fomites, or contaminated water sources [6]; hence, understanding the wild waterfowl host factors responsible for the dissemination of AIV is crucial for improving disease management.

Individual heterogeneity in infectiousness is considered to be a driving force in the development of infectious disease epidemics [7], with high shedding individuals thought to be key in enhancing outbreak intensity [8, 9]. Birds infected with RNA viruses, including LPAIV-infected mallards (*Anas platyrhynchos*), are observed to shed virus with high heterogeneity, where 20% of the birds shed 80% of the total virus shed by all birds [10]. While this pattern in infectiousness has been observed and hypothesized to contribute to the dynamics of disease transmission, we know little about what drives this variation.

The intestines and bursa of Fabricius are important sites for LPAIV replication in wild waterfowl [11–13]. Most LPAIVs circulating in waterfowl preferentially bind to glycans tipped with sialic acid bound to galactose (Gal) in an α-2,3 position (SAα2,3Gal) [14, 15]. These receptors found on epithelial cells are throughout the bird’s respiratory tract, intestinal tract [16–18], and bursa of Fabricius [13]. In birds, the nucleoprotein antigen for LPAIV has most frequently been detected in the intestines and bursa [12, 13, 19]. Additionally, LPAIV-infected birds have more virus isolated from cloacal swab samples than oropharyngeal swabs [20]. Therefore, the distribution and abundance of these receptors in avian intestines and bursa are likely to determine the host’s susceptibility to infection and the virus’s ability to replicate.

Similar to the observation of individual heterogeneity in mallard viral load, variation in sialic acid receptor expression has also been observed. In 76 avian species assessed, 20% of them expressed 80% of the sialic acid receptors observed on erythrocytes in all species [21]. Similarly, 20% of 340 birds expressed 80% of the sialic acid receptors expressed on erythrocytes in all birds assessed [21]. Individual variation of SAα2,3Gal expression in mallard intestines has been observed with some individuals having lower expression of SAα2,3Gal in the ileum, cecum, colon, and bursa compared to other individuals [13]. Differences in the distribution and intensity of SAα2,3Gal between wild bird species have also been observed, such as red head ducks (*Aythya Americana*), black swans (*Cygnus atratus*), and northern pintails (*Anas acuta*) having limited SAα2,3Gal expression in the duodenum and jejunum compared to other Anseriformes [18]. Variation was also found within species, such as mallards, based on the lectin used, *Maackia amurensis* I (MAL I) vs. *Maackia amurensis* II (MAL II) [18]. While previous literature suggests there is variation in SAα2,3Gal abundance and distribution within and across species, the occurrence frequency of SAα2,3Gal in the intestines and bursa has yet to be statistically quantified and related to LPAIV load, a first step in understanding this potential source of AIV variability across individuals and species.

In this study, we address this knowledge gap by investigating the relationship between SAα2,3Gal and LPAIV load in mallards and blue-winged teals (*Spatula discors,* hereafter referred to as “teal”). Both species are important hosts for LPAIV. The mallard is important because of their worldwide distribution, their periodomesticity, and the large diversity of AIV strains isolated from them, including highly pathogenic strains causing high mortality in poultry and people [3, 22, 23]. Teals have high infection prevalence [24] and an important role in over-wintering the virus in the southern United States [25, 26].

We hypothesized that a higher occurrence frequency of SAα2,3Gal in mallards and teals corresponds with higher LPAIV titers. Additionally, we hypothesized that the relationship between virus titers in cloacal swab, ileum tissue, and bursa tissue would all be positively related to each other. Sex-based differences, species-based differences, and comparisons in the occurrence frequency of SAα2,3Gal between control and infected birds was also analyzed, where we did not expect to see differences. This research provides a first look into this putative intrinsic factor responsible for LPAIV individual variation in mallards and blue-winged teals.

## Results

### Distribution of birds in experimental groups

**Fig. 1:**
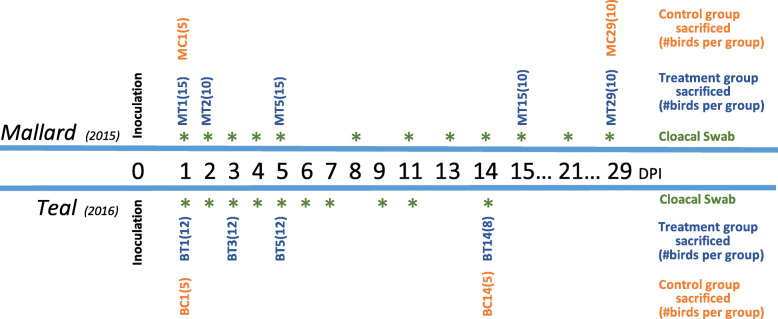
Timeline of Sample Collection for Mallards and Blue-winged Teals. Experimental groups are designated by species (M = mallard, B = blue-winged teal), infection status (T = LPAIV treatment/inoculation with LPAIV H5N9, C = control/sham-inoculated), and day post infection (DPI) the group of birds was sacrificed. Two mm sections of bursa of Fabricius tissue and ileum tissue were collected from each bird on the DPI of sacrifice. Cloacal swabs were collected from all living birds at each DPI designated by an asterisk

Mallards (*n* = 70) and teals (*n* = 54) were assigned to LPAIV treatment (inoculated with LPAIV H5N9) and control groups (sham-inoculated) prior to LPAIV inoculation and sample collection (cloacal swab, ileum tissue, and bursa of Fabricius tissue; Fig. 1). Birds in both treatment groups and control groups were assigned to smaller groups based on the day post infection (DPI) they were sacrificed. Body mass (mallard: range = 640 to 1020 g, mean = 849 g; teal: range = 285 to 473 g, mean = 362 g), age (mallard: range = 60 to 120 days, mean = 87 days; teal: range = 64 to 86 days, mean = 76), and sex (mallard: male = 34, female = 36; teal: male = 26, female = 28) were equally distributed across experimental groups.

### Viral infection of mallards and teals

All birds inoculated with LPAIV H5N9 (mallard = 60, teal = 44) were infected as demonstrated by detection of LPAIV RNA (qPCR Ct values < 40) in cloacal swabs, ileum tissue, and/or bursa tissue collected during the first five DPI (Additional File 1). No birds shed virus past 15 DPI, and of the birds that survived to 15 DPI, 99.9 (mallard) and 98.5 (teal) percent of the total virus shed by those birds occurred in the first five DPI. As expected with LPAIV, we observed no clinical signs of disease such as ruffled feathers, lethargy, respiratory distress, or any pathology.

### Relationship of virus titers in cloacal swab, ileum tissue, and bursa of Fabricius tissue

**Fig. 2:**
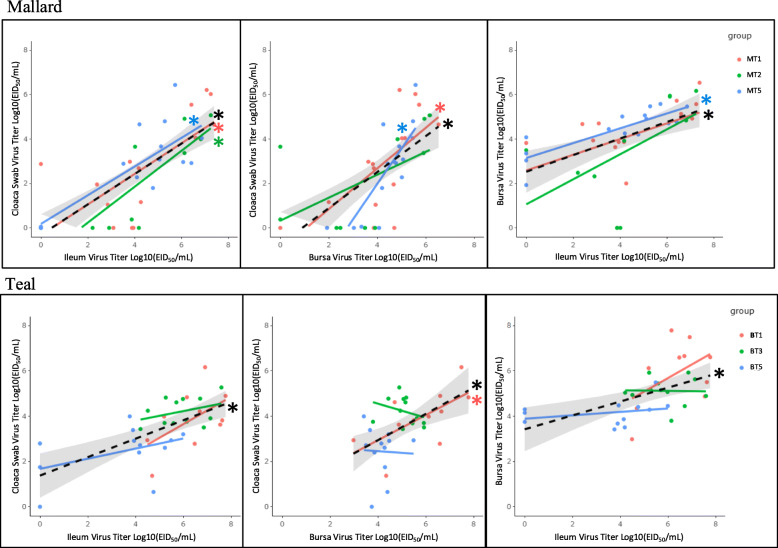
LPAIV H5N9 virus titers in the bursa, ileum, and cloacal swabs are positively related. Black trendline is the linear regression for all birds sacrificed on 1–5 days post infection (DPI) with the 95% confidence interval shaded in gray. Colored trendlines represent each treatment group: T1 represents birds sacrificed on 1 DPI, etc. Trendlines indicated with a (*) indicate a statistically significant relationship (*p* < 0.05)

Statistically significant (*p* < 0.05) positive linear relationships were observed between virus titers in cloacal swabs collected at DPI of sacrifice, ileum tissue, and bursa tissue for mallard LPAIV treatment groups MT1, MT2, MT5 and teal LPAIV treatment groups BT1, BT3, BT5 (Fig. 2). In mallards, statistically significant positive relationships were observed between ileum virus titers and cloacal swab virus titers for all LPAIV treatment groups (MT1, slope parameter estimate (Est.) = 0.69, R^2^ = 0.43, *p* = 0.005; MT2, Est. = 0.81, R^2^ = 0.64, *p* = 0.003; and MT5, Est. = 0.65, R^2^ = 0.66, *p* < 0.001). Statistically significant positive relationships were observed between bursa virus titers and cloacal swab virus titers for LPAIV treatment groups MT1 (Est. = 0.92, R^2^ = 0.41, *p* = 0.006) and MT5 (Est. = 1.60, R^2^ = 0.63, *p* < 0.001). Only MT5 (Est. = 0.33, R^2^ = 0.68, *p* < 0.001) had a statistically significant positive relationship between ileum virus titers and bursa virus titers. In teals, the only statistically significant positive relationship for LPAIV treatment groups was observed for BT1 (Est. = 0.56, R^2^ = 0.34, *p* = 0.036) between cloacal swab virus titers and bursa virus titers.

### Species and sex-based differences in viral shedding

**Fig. 3:**
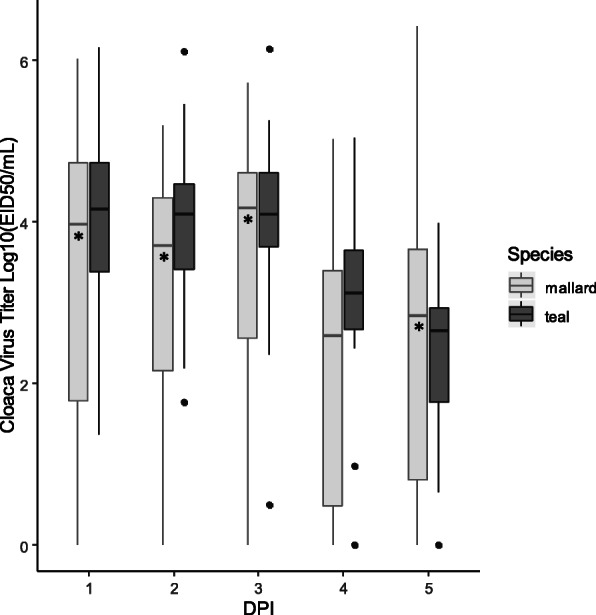
Cloacal swab virus titer boxplots for mallard and blue-winged teals infected with LPAIV H5N9. Horizontal bar within the box is the median value, solid dots indicate values falling above the upper or below the lower quartile + 1.5 times the interquartile distance. (*) indicates statistically higher variation between species for each day post infection (DPI; *p* < 0.05)

Looking at all virus titers from cloacal swab samples collected from LPAIV treatment groups in the first five DPI, statistically significant differences were found between mallards and teals, but not between males and females within species. Mallards had statistically higher variation than teals in cloacal swab viral titers on one, two, three, and five DPI (Fligner-Killeen *p* < 0.05; Table 1, Fig. 3). For both species, mean cloacal swab virus titers on one, two, and three DPI were statistically higher than virus titers on four and five DPI (F_4,242_ = 17.61, *p* < 0.001; Additional File 2). Teals shed statistically more virus than mallards (F_1,102_ = 14.60, *p* < 0.001) with no interaction between species and DPI (F_4,242_ = 0.91, *p* = 0.456; Additional File 2). No sex-based differences were observed in cloacal swab virus titers for either species (mallard: F_1,58_ = 0.05, *p* = 0.818; teal: F_1,42_ = 2.49, *p* = 0.122) with no statistically significant interaction between sex and DPI (mallard: F_4,138_ = 0.39, *p* = 0.818; teal: F_4,96_ = 2.43, *p* = 0.053; Additional File 3).

**Table 1:** Virus titer descriptive statistics for mallard and blue-winged teal cloacal swabs

DPI	Species	N	N > DL	N > QL	min +	max	mean	std.dev
					Log10(EID50/mL)	Log10(EID50/mL)	Log10(EID50/mL)	Log10(EID50/mL)
**DPI 1**	mallard	58	52	38	0.13	6.21	3.26	*1.94
	teal	44	44	41	1.37	6.16	4.06	0.99
**DPI 2**	mallard	43	38	28	0.38	5.2	3.11	*1.64
	teal	32	32	29	1.78	6.12	3.96	0.97
**DPI 3**	mallard	35	29	26	0.06	5.72	3.33	*1.90
	teal	32	32	29	0.5	6.15	4.05	0.98
**DPI 4**	mallard	35	29	19	0.19	5.03	2.38	1.54
	teal	20	19	15	0.98	5.04	3.08	1.16
**DPI 5**	mallard	35	28	18	0.05	6.43	2.29	*1.75
	teal	20	19	10	0.65	3.99	2.37	1.01

*N* total sample size, *DL* detection limit of 0.04 Log10(EID_50_/mL), *QL* quantification limit of 2.60 Log10(EID_50_/mL), *min +* minimum N > DL, mean is the geometric mean, and std.dev = one standard deviation. (*) signifies significantly higher titer variation for each DPI between species

### Evaluating SAα2,3Gal in intestines and bursa of Fabricius

**Fig. 4:**
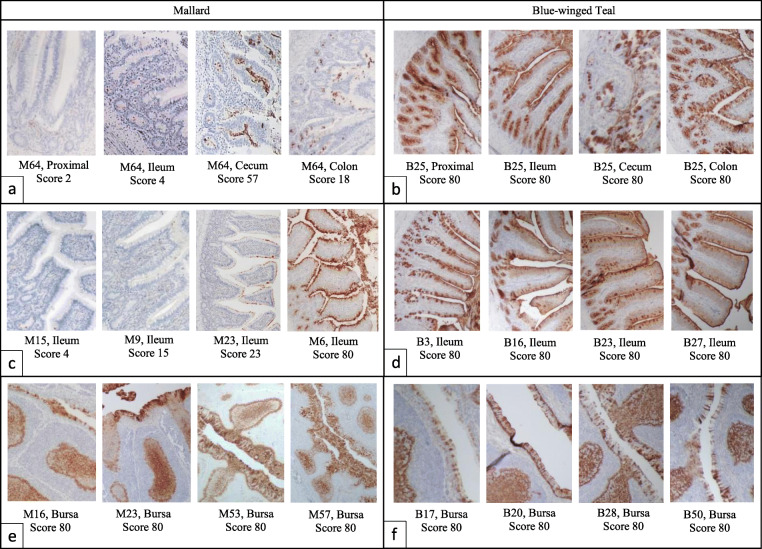
Lectin staining of mallard and blue-winged teal intestines and bursa of Fabricius. Lectin binding is positive where the brown colored stain is visible. The individual bird ID, tissue, and lectin score (villi enterocyte/epithelial cells) are given for each histological photograph. Scores were determined by averaging the scores for each field of view evaluated at 400x. Each field of view was given the following score: 0, no cells stained; 5, 1–10% of cells were stained; 35, 11–60% of cells stained; and 80, 61–100% of cells stained. Segments (a) and (b) show the range of lectin scores between sections of intestinal tissue in one individual (proximal represents duodenum or jejunum). Segments (c) and (d) show the range of lectin between individuals for the ileum tissue specifically. Segments (e) and (f) show lectin scores in the bursa of Fabricius. All photos were taken at 200x brightfield microscopy

Frequency of SAα2,3Gal occurrence in the intestines and bursa of Fabricius was determined by visually assessing MAL I lectin stained cells and assigning a “lectin score” based on the estimated percentage of cells stained in each microscopic field of view (400x) per tissue sample. Initial observation of lectin staining in mallard intestinal tissues revealed incongruent staining of the intestinal brush border, villi enterocytes, and crypt enterocytes; therefore, these three “cell types” of the duodenum, jejunum, ileum, cecum, and colon of each bird were assessed separately and received their own lectin score (Fig. 4, Additional File 1). The majority of mallard bursa epithelial cells were autolyzed, thus the lectin score for mallard bursa was not evaluated. Autolysis also affected 5.7% (85/1488) of intestinal tissue/cell types assessed. Any individual tissue/cell type that could not be scored was removed from analysis.

### Lectin score differences between infected and control birds

**Fig. 5:**
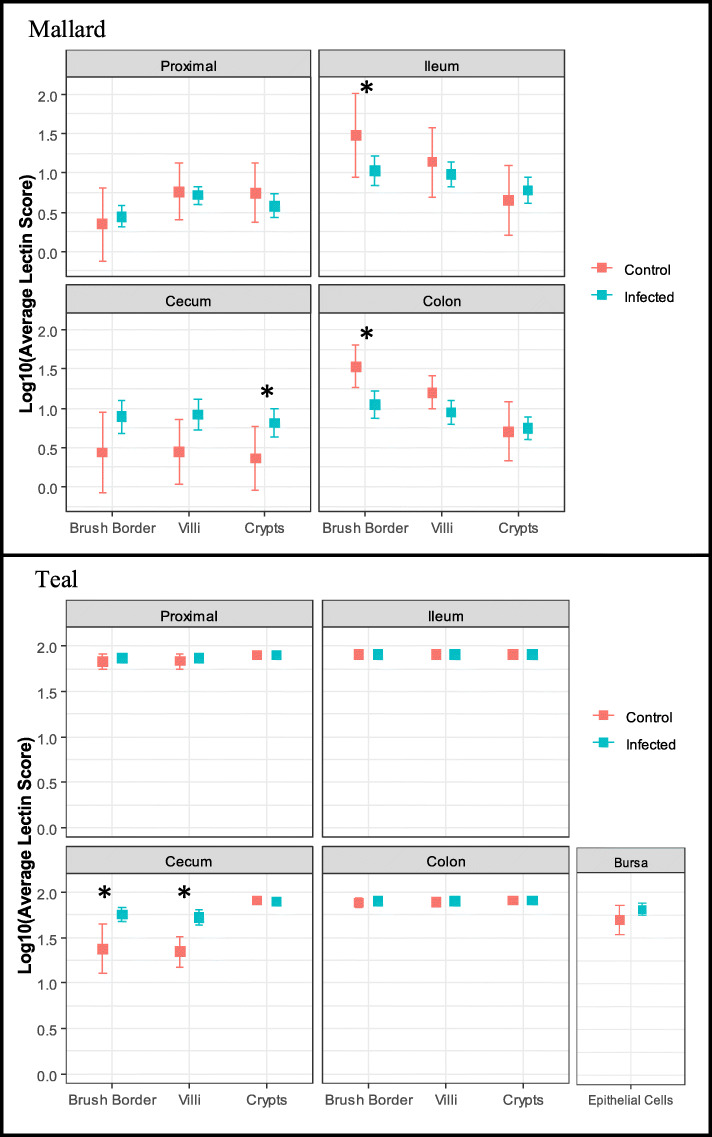
Lectin score differences between control and LPAIV-infected birds. Mean lectin scores + 95% confidence intervals of intestinal tissues proximal (duodenum and jejunum), ileum, cecum, and colon for LPAIV H5N9 infected and control mallards and blue-winged teals. Bursa epithelial cells are included for teals only. (*) indicates tissue/cell type with a statistically significant difference between control and infected birds (*p* < 0.05)

Analyzing mallards and teals in two separate statistical models, lectin scores were not statistically different between LPAIV treatment and control mallards (F_1,68_ = 0.11, *p* = 0.746); however, there was a statistically significant interaction between infection status and tissue/cell type (F_11,693_ = 4.08, *p* < 0.001). We found the cecum crypt lectin score in LPAIV treatment mallards to be statistically higher than control mallards (*p* = 0.046; Fig. 5). Conversely, lectin scores of control mallards’ ileum brush border (*p* = 0.017) and colon brush border (*p* = 0.015) were statistically significantly higher than LPAIV treatment mallards (Fig. 5). Unlike mallards, LPAIV treatment teals had statistically higher lectin scores than control teals (F_1, 52_ = 15.20, *p* < 0.001), with a statistically significant interaction between infection status and tissue/cell type (F_12,611_ = 8.66, *p* < 0.001). Post-hoc analysis shows the lectin score in the cecum brush border (*p* < 0.001) and cecum villi (*p* < 0.001) was higher in LPAIV treatment birds than control birds (Fig. 5).

### Lectin score species and sex-based differences

**Fig. 6:**
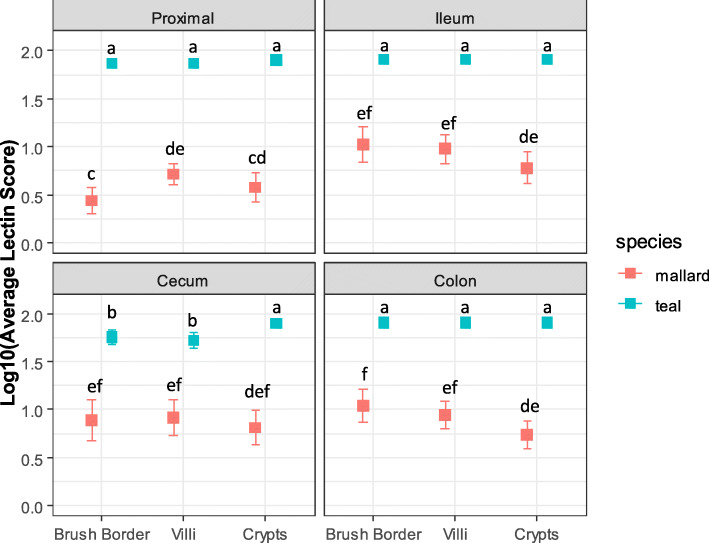
Lectin score differences between mallard and blue-winged teal intestinal tissues. Mean lectin scores + 95% confidence intervals for intestinal tissues proximal (duodenum and jejunum), ileum, cecum, and colon for LPAIV H5N9 infected mallards and blue-winged teals. Across all panels, points with different letters are considered significantly different (*p* < 0.05)

Looking at birds only in LPAIV treatment groups, higher inter-tissue and inter-individual variation was observed in mallards compared to teals for all tissue/cell types (Fligner-Killeen *p* < 0.001; Table 2; Fig. 4). LPAIV treatment teals had statistically higher lectin staining than LPAIV treatment mallards (F_1,102_ = 309.92, *p* < 0.001) with a statistically significant interaction between species and tissue/cell type (F_11,1067_ = 9.95, *p* < 0.001). In mallards, the ileum, cecum, and colon had statistically similar lectin scores for most cell types; however, lectin scores for most cell types in the proximal intestine were significantly lower (*p* < 0.05) than the lectin scores in ileum, cecum, and colon (Fig. 6). In teals, most tissues/cell types had similar lectin scores, except for the cecum brush border and cecum villi, which were statistically significantly lower than all other tissue/cell types (Fig. 6).

**Table 2:** Lectin histochemistry score descriptive statistics

Tissue	Cell Type	Species	N	min (%)	max (%)	mean (%)	std.dev (%)
Proximal	crypts	teal	44	44.25	80.00	79.19	5.39
		mallard	60	0.00	80.00	10.22	*19.92
	brush border	teal	44	40.25	80.00	74.24	12.12
		mallard	60	0.00	80.00	6.91	*16.99
	villi	teal	44	40.55	80.00	74.46	12.12
		mallard	60	0.00	80.00	8.82	*16.12
Ileum	crypts	teal	44	80.00	80.00	80.00	0.00
		mallard	54	0.00	80.00	14.51	*22.26
	brush border	teal	43	80.00	80.00	80.00	0.00
		mallard	47	0.00	80.00	23.36	*27.30
	villi	teal	43	80.00	80.00	80.00	0.00
		mallard	47	0.00	80.00	16.87	*20.47
Cecum	crypts	teal	44	38.00	80.00	78.82	6.47
		mallard	58	0.00	80.00	18.97	*27.25
	brush border	teal	43	7.00	80.00	62.96	23.49
		mallard	54	0.00	80.00	26.24	*33.35
	villi	teal	43	8.00	80.00	59.21	24.95
		mallard	54	0.00	80.00	23.12	*30.47
Colon	crypts	teal	44	80.00	80.00	80.00	0.00
		mallard	59	0.00	80.00	11.66	*19.36
	brush border	teal	44	76.00	80.00	79.91	0.60
		mallard	58	0.00	80.00	23.55	*24.91
	villi	teal	44	76.00	80.00	79.91	0.60
		mallard	58	0.00	80.00	16.35	*18.98
Bursa	Epithelial Cells	teal	42	10.00	80.00	68.57	20.61
		mallard	NA	NA	NA	NA	NA

Proximal includes duodenum and jejunum. *N* total sample size and std.dev = one standard deviation. (*) signifies significantly higher lectin score variation for each tissue/cell type between species

Analyzing LPAIV treatment mallards and teals in separate models, we found that lectin staining was not significantly different between males and females (mallard: F_1,58_ = 2.243 *p* = 0.141; teal: F_1,42_ = 0.24, *p* = 0.626) for either species, and there was no significant interaction between sex and tissue/cell type (mallard, F_11,587_ = 1.48, *p* = 0.136; teal, F_11,458_ = 0.42, *p* = 0.947; Additional File 4).

### Relationship between lectin score and virus titer – mallard

With 99% of positive virus titers on 1–5 DPI, mallards in LPAIV treatment groups MT1, MT2, and MT5 were used in each model to evaluate the association between virus titers and lectin scores. Because lectin scores could not be obtained for mallard bursa tissue due to autolysis, only cloacal swab virus titers and ileum tissue virus titers were analyzed. Missing intestinal lectin scores due to autolysis reduced the sample size for each model from 40 to 25 birds (MT1 = 6; MT2 = 8; MT5 = 11). High correlations (Pearson’s r > 0.8) between lectin scores for proximal brush border, villi enterocytes, and crypt enterocytes, as well as between lectin scores for cecum brush border, villi enterocytes, and crypt enterocytes were observed; therefore, singular variables (proximal PC, cecum PC) for each respective tissue were created using principal component analysis (PCA; Additional File 5).

For cloacal swab virus titers on the DPI of sacrifice, initial stepwise variable selection rendered a multiple linear regression (MLR) model which included sex, proximal PC, ileum villi, and ileum brush border (AIC = 11.44, ΔAIC = 1.77; Additional File 6). This reduced model was tested for co-linearity issues and residual plots were evaluated with no serious statistical problems detected (Additional File 6), so the reduced model was selected as the best fitting model (R^2^ = 0.66, *p* < 0.001; Table 3). Our results show that lectin staining in the ileum villi and being male were positively associated with a higher virus titer, while lectin staining in the ileum brush border was negatively associated with a higher virus titer. Lectin staining in the proximal intestine was not a significant term in the model.

**Table 3:** Sex and ileum lectin scores are associated with LPAIV H5N9 virus titers in mallards

Y	*N*	R^2^	X	Est. (95% CI) Log10(EID50/mL)	*P*
Mallard Cloaca Swab Virus Titer	25	0.66	Intercept	1.37 (0.14 to 2.60)	0.031
			Sex (Male)	1.66 (0.60 to 2.73)	0.004
			Proximal PC1	0.50 (−0.22 to 1.22)	0.166
			Ileum Villi	2.93 (1.42 to 4.44)	< 0.001
			Ileum Brush Border	−1.96 (−3.12 to −0.80)	0.002
Mallard Ileum Virus Titer	25	0.33	Intercept	2.86 (1.21 to 4.52)	0.002
			Sex (Male)	1.36 (−0.19 to 2.92))	0.083
			Ileum Villi	3.27 (1.18 to 5.36)	0.004
			Ileum Brush Border	−1.93 (−3.73 to −0.14)	0.036

*Y* dependent variable, *N* number of individual birds in model, *X* independent variables in final model, *CI* 95% confidence interval, *p* p-value. Dependent variable “mallard cloaca virus titer” includes virus titers from cloacal swabs collected on the DPI each bird was sacrificed. Proximal includes the duodenum and jejunum. PC1 represents the principal component variable for the proximal villi enterocytes, brush border, and crypt enterocytes combined. BCS = Body Condition Score. Group and Sex were treated as factors in each model, and if present in final model, group T1 and females are represented in the intercept

For mallard ileum virus titer, initial stepwise variable selection rendered a model which included sex, ileum villi, and ileum brush border (AIC = 33.57, ΔAIC = 1.32; Additional File 7). This reduced model was tested for co-linearity issues and residual plots were evaluated with no serious statistical problems detected (Additional File 7), thus this model (R^2^ = 0.33, *p* < 0.010, Table 3) was selected as the best fitting model. Our results show that the lectin score of the ileum villi was positively associated with a higher virus titer. The lectin score in the ileum brush border was negatively associated with a higher virus titer. Sex was not a significant factor in this model.

### Relationship between lectin score and virus titer – teal

With 98% of positive virus titers on 1–5 DPI, teals in LPAIV treatment groups BT1, BT3, and BT5 were used in each model to evaluate the association between virus titers and lectin scores. Missing lectin scores due to autolysis reduced the sample size for each model from 36 to 32 birds (T1 = 9, T3 = 11, T = 12).

For cloacal swab virus titers on the DPI of sacrifice, initial stepwise variable selection rendered a model which included sex, mass, body condition score (BCS), LPAIV treatment group, proximal crypts, and bursa (AIC = − 8.31, ΔAIC = 0.10; Additional File 8). This reduced model was tested for co-linearity issues and residual plots were evaluated with no serious problems detected, thus this model (R^2^ = 0.61, *p* < 0.010) was selected as the best fitting model (Additional File 8); however, inconsistent results were observed when validating results with respect to quantification limit assumptions (Additional File 9). Due to these inconsistencies, we conclude the model to be unstable and results unreliable.

For teal ileum virus titer, initial stepwise variable selection rendered a model which included BCS and LPAIV treatment group (AIC = 32.41, ΔAIC = 1.99; Additional File 10). This reduced model was tested for co-linearity issues and residual plots were evaluated with no serious problems detected, thus this model (R^2^ = 0.44, *p* < 0.001) was selected as the best fitting model (Additional File 10). Our results show that virus titers were lower on five DPI compared to one and three DPI. BCS was not a significant term in the model.

For teal bursa virus titer, initial stepwise variable selection rendered a model which included mass, BCS, and treatment group (AIC = − 1.6, ΔAIC = 1.75; Additional File 11). The reduced model was tested for co-linearity issues with no problems detected. Residual plots were evaluated, and the model did not fit normality assumptions. Mass was removed from the model since it was an insignificant factor, and the residual plots improved; therefore, the model which included BCS and LPAIV treatment group was accepted as the best fitting model (R^2^ = 0.37, *p* = 0.001; Additional File 11). Our results show that virus titer was highest on one DPI, and significantly lower on three and five DPI. BCS was not a significant term in the final model.

## Discussion

Mallards and blue-winged teals are important reservoir hosts for avian influenza viruses [3, 24, 25]; they are both widely distributed waterfowl species and commonly infected with both LPAIV and HPAIV. Our study documents both within and between-species variation in viral shedding and occurrence frequency of SAα2,3Gal, the viral receptor for many LPAIVs. In mallards, but not teals, we found viral shedding was related to lectin scores, which represent the occurrence frequency of SAα2,3Gal. While we expected to see positive linear relationships between virus titers and SAα2,3Gal in all tissues and cell types, the mallard ileum was the most predictive of virus titers, with a positive relationship between virus titers and SAα2,3Gal in ileum villi enterocytes, and a negative relationship between virus titers and SAα2,3Gal in the ileum brush border. Despite the lack of relationship between viral shedding and SAα2,3Gal in teals, we observed significantly higher viral shedding by teals, and a higher occurrence frequency of SAα2,3Gal compared to mallards.

As the direction (positive or negative) of the correlation between SAα2,3Gal occurrence frequency and virus titer varied across mallard tissue locations, our data highlight the importance of understanding tissue-specific tropism as it relates to cell surface SAα2,3Gal distribution. Within mallards, the positive relationship between virus titer and SAα2,3Gal in the ileum villi enterocytes was expected given that LPAIV replicates in intestinal enterocytes by binding SAα2,3Gal on the surface of the cell for cell entry [27]. A reason ileum villi enterocytes were most correlated with viral titer compared to ileum crypt enterocytes may be that the villi have closer direct contact with digesta and as a result, closer direct contact with virus passing through the gut. For example, previous studies have found LPAIV antigen via immunohistochemistry more consistently in mallard villi enterocytes compared to the crypts [12, 13].

Two hypotheses could explain the negative relationship between SAα2,3Gal in the ileum brush border and virus titer. Initially, we expected to see a positive relationship between SAα2,3Gal in the brush border of all intestinal tissues and virus titers since the receptors are on the surface of the cell and more likely to be exposed to virus [28]. However, as a virion attaches to a receptor, the virion along with the receptor becomes engulfed by the cell for replication, therefore removing the receptor from the surface of the cell [29]. This idea is also consistent with the differences observed in occurrence frequency of SAα2,3Gal between LPAIV treatment and control mallards, where control mallards had higher SAα2,3Gal in the ileum and colon brush border compared to LPAIV treatment birds. Second, mucus is also found along the brush border and LPAIV has been found to bind SAα2,3Gal in mucus, which would prohibit the virus from reaching the enterocyte for virus replication [15, 30, 31]; thereby reducing the quantity of virus shed. Up-regulation of mucins have also been observed in response to other viruses which bind sialic acid receptors [32], such as human rotavirus infections [33]. To understand the true source for the negative relationship between occurrence frequency of SAα2,3Gal in the ileum brush border and virus titers, further experimental research is warranted.

Our results do not show a relationship between virus titer and SAα2,3Gal occurrence frequency in the other three intestinal tissue types: proximal, cecum, and colon. The lack of a statistically significant relationship between SAα2,3Gal and virus titer in the mallard colon was unexpected, given many studies have showed the colon as a site for high LPAIV replication [11–13, 34]. Since SAα2,3Gal in the ileum and colon were 63% correlated with each other (Additional File 12), the colon could also have a contributing effect to viral load, but not as strongly as the ileum. Because the cecal tonsils, a major lymphoid tissue in the cecum, enlarge during gut infections due to infiltration of immune cells [35], perhaps a relationship between virus titer and SAα2,3Gal in the cecum could not be detected because of the interference of immune cells which may have been identified as enterocytes when stained. SAα2,3Gal in the mallard proximal intestine did not show a relationship with virus titers likely because we observed a lower frequency of SAα2,3Gal in the proximal intestine compared to the ileum, cecum, and colon. Previous findings show that positive viral antigen is more commonly found in the ileum, cecum, and colon when cloacal swab virus titers are high [12, 13], which would suggest that the proximal intestine is not a prime site of LPAIV replication. While we did not detect statistically significant relationships between virus titers and SAα2,3Gal in the proximal intestine, cecum, or colon, we cannot say for certain these tissues do not contribute to viral shedding. Our results indicate, however, that ileum SAα2,3Gal occurrence frequency has the strongest relationship to viral load in mallards.

The bursa epithelial cells are also considered an important site of replication for LPAIV in waterfowl, including mallards [12, 13]. However, given autolysis of tissue samples, we could not analyze the relationship between SAα2,3Gal in the bursa and viral shedding in mallards. In teals, lectin staining was very high in the bursa; however, it was not significantly related to viral shedding. Lack of a significant relationship to viral titer in teals could be attributed to the lack of individual variation in SAα2,3Gal expression in the bursa or to a sporadic correlation between bursa and cloacal swab virus quantity. Further analysis of bursa sialic acid receptors is therefore warranted to determine relationships with LPAIV viral load.

Although we did not determine a linear relationship between SAα2,3Gal and virus titers in blue-winged teals, significantly higher virus titers and a higher occurrence frequency of SAα2,3Gal with less variation were observed in teals compared to mallards. We hypothesize that the higher teal virus titers resulted from higher SAα2,3Gal occurrence frequency. Teals have already been shown to have a higher binding affinity to MAL I lectin than mallards [18]. Different LPAIV strains also vary in binding affinity to SAα2,3Gal with different molecular structures [36]. Although, LPAIV H5N9 (Ratite/New York/12716/94) has a similar affinity for the receptors targeted by MAL I [36, 37], we did not test the specific receptor affinity of the LPAIV H5N9 (A/northern pintail/California/44221–761/2006) used in this study. If LPAIV H5N9 (A/northern pintail/California/44221–761/2006) has a higher affinity for SAα2,3Gal with a β1-4Glc(NAc) linkage, the preferred binding affinity of MAL I, then our results provide further evidence to explain the higher LPAIV H5N9 virus titers in teals. However, the converse is at least theoretically possible; that is, higher receptor abundance was a result rather than a cause of higher viral titers in teal. Receptor abundance would have to be assayed prior to and during viral infection to disentangle these issues, which is a significant experimental hurdle.

Species-based variation in SAα2,3Gal has been observed in other experimental infection studies [18, 21]. Jankowski et al. [21] analyzed the variation of sialic acid receptors expressed by erythrocytes in various avian species and found that approximately 20% of the species expressed 80% of the overall sialic acid receptor quantity in all species studied. Although teals were not included in the Jankowski et al. [21] study, mallards and three other *Anas* species (*A. acuta, A. Americana, and A. crecca*) were among the species assessed. Interestingly, mallards had the lowest quantity of sialic acid receptors on erythrocytes compared to the other three *Anas* species. Our results which show mallards with lower frequencies of SAα2,3Gal compared to teals provide further evidence of species-based differences in sialic acid receptors.

The premise of our study was to determine if the occurrence frequency of SAα2,3Gal in the intestines and bursa may be associated with cloacal shedding; hence, we predicted the variation of SAα2,3Gal in control and infected birds would not differ. Our data suggest this is not the case. In the cecum, the occurrence frequency of SAα2,3Gal was higher in the crypts of infected mallards compared to their conspecific controls. Similarly, in teals the frequency of SAα2,3Gal was higher in the cecum villi and brush border of infected birds. The ceca have a unique role in the functioning of the vertebrate immune system. As stated previously, the cecal tonsils, a major lymphoid tissue in the cecum, enlarge during gut infections due to infiltration of immune cells, which also includes macrophages [35]. Macrophages express Gal-specific receptors [38], which could explain the higher abundance of SAα2,3Gal in the cecum of infected birds relative to controls. Evidence of macrophages expressing Gal-specific receptors are seen in white leghorn chickens, which in one study had a greater abundance of sialic acid receptors than silky fowl because of a higher number of immune cells in the leghorns’ cecum [39]. The cecum has a unique response to LPAIV infection compared to other intestinal tissues, which warrants further analysis of SAα2,3Gal in this tissue.

Contrary to differences in SAα2,3Gal expression between LPAIV-infected and control birds in the cecum, control mallards expressed more SAα2,3Gal in the ileum and colon brush border than infected mallards. Franca et al. [13] found that SAα2,3Gal was lower in the cecum, colon, and bursa of infected birds compared to control birds. Their hypothesis indicated that the SAα2,3Gal expression level may decrease after infection because the neuraminidase function of the virus allows cleaving of the receptor releasing virions from the cell [40]. When the receptor is cleaved, it is no longer present on the cell surface which would reduce lectin binding. While Franca et al. [13] did not specify whether the decrease in lectin staining was on the surface of the enterocyte, we found mallards to have a higher occurrence frequency of SAα2,3Gal only in the brush border. Our results indicate the importance of assessing the specific location of SAα2,3Gal in determining their function in influenza studies.

No difference was detected between males and females in virus titers or frequency of SAα2,3Gal when examined separately in either species; yet, when SAα2,3Gal in the ileum villi enterocytes and brush border are held constant, a statistically significant difference in cloacal swab virus titer was detected between male and female mallards. Biologically, our results show that due to the natural variation of SAα2,3Gal frequency in the ileum of mallards, sex is not important to the viral shedding variation observed in the population; however, it may be a contributing factor in the relationship between viral load and SAα2,3Gal frequency in the ileum. The unique relationship between sex, SAα2,3Gal in the ileum, and cloacal swab virus titers in mallards warrants further research for understanding why sex would be important for the relationship between viral load and SAα2,3Gal in the mallard ileum.

The identified positive relationships between viral RNA in cloacal swabs, ileum tissue, and bursa tissue further supports the importance of the ileum and bursa for cloacal shedding of LPAIV. Prior to this study, it was well known that LPAIV replicates in duck intestines and the bursa of Fabricius [11–13]. While testing for virus in cloacal swabs is the standard method for determining AIV fecal shedding [20, 41], the direct relationship between tissue replication and virus shed by the cloaca was unknown. Through quantifying viral RNA via qPCR in ileum and bursa tissue, significant positive relationships were found between virus titers in cloacal swabs, ileum tissue, and bursa tissue, showing the contribution of these tissues to the cloacal virus shed. The positive relationship between virus titers in the ileum and cloacal swabs provides additional evidence to support our conclusion that ileum SAα2,3Gal was associated with virus titer. These positive relationships add validity to collecting cloacal swabs as an indicator of virus titer in the ileum and bursa and perhaps the infection status of individual birds.

## Conclusion

Understanding the mechanism underlying variation in infection severity and viral shedding can provide insight into why a few individuals in a population are more infected than others, and perhaps, why some species are more infectious than others. LPAIV is a gut-associated pathogen in wild waterfowl; hence, the physiology of the host’s gut is an important determinant of within-host-pathogen interaction. Our results provide evidence that sialic acid receptors in the gut are associated with viral load. Since sialic acid expression varies both between species [18, 21] and within species [13], this variation has implications for a species’ and/or individual bird’s contribution to the transmission of avian influenza virus. Furthermore, sialic acid is the cellular receptor for other viruses such as parainfluenza, mumps, corona, noro, rota, and DNA tumor viruses, some of which infect humans [33], leading to similar questions regarding the effect of sialic acid receptor variation across individuals and species on host-virus interactions. Pathogen receptors are not the only contributing factor to a host’s infectiousness. Other intrinsic factors and their relationship to pathogen shedding warrant further investigation. Because the quantity of virus shed can directly affect transmission dynamics and is an important parameter for predicting disease risk in a population [42], identifying individuals or certain species as more infectious could improve our ability to predict and mitigate disease.

## Methods

### Permits and protocols

Protocols for animal care and experimental sampling procedures were approved by Michigan State University (MSU) Institutional Animal Care and Use Committee (AUF 12/16–211-00). All euthanasia procedures were in accordance with the Animal Welfare Act and Guidelines to the Use of Wild Birds in Research [43]. Duck eggs were collected with permission from the U.S. Fish and Wildlife Permit (M Bl 94,270–2) and North Dakota Game and Fish Department License #GNF03639403.

### Study species and locations

Mallards and teals used for this study were collected as eggs from the nests of wild birds in the southwest corner of Towner County, North Dakota, USA (48.4431853, − 99.3156225). In May–June 2015 we collected 90 mallard eggs (1–2 per nest) from a total of 50 nests, with each nest containing an average of eight eggs per clutch. The following summer, May – June 2016 we collected 80 blue-winged teal eggs (1–2 per nest) from a total of 40 nests. Nests were found and eggs collected by dragging a heavy metal-link chain behind two ATVs driving in parallel which initiated hens to fly off their nests [44]. Eggs were candled in field to determine age, and any eggs that either had not started incubation or were between 15 and 22 days of incubation were shipped overnight to MSU in East Lansing, Michigan. Each year we made 2–4 shipments of 15 to 40 eggs each over a period of 6 weeks. Unless specified otherwise, all procedures were the same for each species/year.

Upon arrival at MSU, eggs were immediately placed into a climate-controlled egg incubator (Sportsman 1502 Egg Incubator, GQF Manufacturing Co., Savannah, GA) housed within a biosafety level two room within the MSU Research Confinement Facility. Eggs were incubated at 37.5 °C with 45–50% humidity and rotated electronically 10 times per day. Eggs were candled for viability and age once every three days. As soon as eggs pipped, they were moved into a hatching incubator (Sportsman 1502 Egg Incubator, GQF Manufacturing Co., Savannah, GA) at 37.2 °C with 70–80% humidity. Chicks remained in the hatcher until they were dry, approximately 12–24 h post hatching. Each bird was then weighed to the nearest 0.1 g, banded with a uniquely numbered plastic leg band, and placed in a brooder (30–35 °C). Birds were kept in brooders for two weeks, then moved to open-room housing where a maximum of 35 birds were housed per room (400sq feet). Each room maintained a temperature of 23 °C and 45–55% humidity, had two swimming pools (45″ diameter, 10″ depth), and two dry pools with aspen chip bedding. In both years, birds were maintained on a 13:11 h light:dark photoperiod.

Birds were fed ad libitum Purina® Flock Raiser® Crumbles (Purina, St. Louis, MO, USA) and supplemented with chopped dandelion greens twice per day. Rooms were fully cleaned twice per day. Birds were routinely checked for normal health and weighed every five days. One week prior to inoculation, mallards were separated into individual cages of 20 cages per room. Blue-winged teals were kept in the open room housing separated by experimental group.

### Virus

LPAIV A/northern pintail/California/44221–761/2006 (H5N9), originally collected from a northern pintail cloacal swab and isolated in specific pathogen free embryonated chicken eggs (ECE), was acquired from the USGS National Wildlife Health Center in Madison, WI (USDA Veterinary Permit 44,372). We prepared stock virus propagating the virus in 9 to 11-day old ECE (Charles River, Norwich, CT, USA) [45]. The infectious titer of the stock virus of 7.63 log EID_50_/ml was determined using the 50% egg infectious dose (EID_50_) and calculated using the Reed & Muench method [46]. The viral inoculum was prepared by diluting the stock virus in Dulbecco’s Modified Eagle Medium (DMEM) (Gibco® by Life Technologies, Grand Island, NY, USA) to yield a final titer of 5.63 log EID_50_/ml.

### Experimental design

Individual birds were assigned to one of two control groups for each species, one of five mallard LPAIV treatment groups, or one of four teal LPAIV treatment groups (Fig. 1). Experimental group assignment was done using pseudo-stratified randomization with birds being stratified by body mass, age, and sex. Additionally, individuals from the same nests were assigned to separate groups. Group names refer to their species (mallard = M, teal = B), whether they received LPAIV treatment (inoculated with virus = T, control = C), and the DPI they were sacrificed (# to follow T/C). The minimum sample size per group was based on individual viral load variation observed in populations as small as 10 individuals [10]. Additional birds were placed in groups on DPI of most importance such as high viral shedding (DPI 1–3) and early detection of antibody titer (DPI 5) [47].

All LPAIV treatment group birds (also referred to as “infected”) were inoculated with 1.0 mL of 5.63 log EID50/ml viral inoculum on 0 DPI, diluted in DMEM by placing one drop on each eye and each nare, then dispensing the rest in the esophagus [48, 49]. All control birds were sham-inoculated with 1.0 mL of sterile DMEM in a similar fashion. During the inoculation and after inoculation, birds were kept in biosafety level two conditions and personal protective equipment consisted of non-vented, full coverage eye goggles, hair cap, N95 respirator, double gloves, tyvek suit, and plastic booties.

We collected cloacal swabs on all live individuals. Cotton tipped swabs were collected from mallards on 1–5, 8, 11, 13, 15, 17, 19, 22, 24, 26, and 29 DPI, and from teals on 1–7, 9, 11, and 14 DPI (Fig. 1). Swabs were stored in 3.0 mL of brain-heart infusion broth (BHI), transported on ice, and stored in − 80 °C until sample processing.

### Euthanasia

Mallards, as described by their assigned groups, were sacrificed on 1, 2, 5, 15, and 29 DPI, and teals were sacrificed on 1, 3, 5, and 14 DPI (Fig. 1). Mallards sacrificed on one DPI were euthanized by intravenous lethal injection of pentobarbital sodium and phenytoin sodium solution (Beuthanasia-D Special, Merck Animal Health, Madison, NJ, USA). All other birds were euthanized by carbon dioxide inhalation. Bird carcasses were preserved on ice until necropsy was performed.

### Necropsy and tissue collection

Mallard necropsy was performed in the same room where birds were kept under biosafety level two conditions mentioned above. Teal necropsies were performed under a biosafety cabinet. Necropsies were performed on mallards within one to six hours of being euthanized, with an average time of approximately four hours post euthanasia. Due to autolysis of tissue samples observed with mallards, we performed necropsies on teals within one hour of being euthanized, with the average time of 22 min post euthanasia. We examined birds for any abnormalities and the coelomic cavity for any gross pathology. We also assessed the birds’ body condition using a scale of one to five: one being emaciated and five being over-conditioned with presence of fat in intestinal mesentery. Sex was determined by examining the syrinx [50].

We collected 0.5 to 2 cm sections of intestine (duodenum, jejunum, ileum, cecum, colon) and bursa of Fabricius in 10% buffered formalin. The tissues were incubated at room temperature for 24–48 h to allow time for fixation, then transferred to a histological sectioning cassette in 70% ethanol and embedded in paraffin within 24 h. We also collected 2 mm sections of ileum and bursa in RNA stabilizing solution (RNAlater®, Sigma-Aldrich, St. Louis, MO, USA) for viral RNA analysis in these tissues.

### Viral RNA isolation and RT-PCR

Virus in cloacal swabs, ileum tissue, and bursa tissue was quantified by isolating viral RNA using qPCR targeting the matrix protein gene [51]. Unlike immunohistochemistry which stains for nucleoprotein antigen, qPCR is quantitative and can detect lower quantities of virus [52]. Viral RNA was isolated from cloacal swab material using the MagMAX™-96 AI/ND Viral RNA Isolation Kit (Applied Biosystems® by Thermo Fisher Scientific, Vilnius, Lithuania) with modifications to the manufacturer protocol previously described [53]. Viral RNA was extracted with host mRNA from 15 to 30 mg of ileum and bursa tissue from each bird using the Qiagen RNeasy Mini Kit (QIAGEN®, Hilden, Germany) according to the manufacturer’s protocol. For the RT-PCR working solution we used the TaqMan® RNA-to-Ct™ 1-Step Kit (Applied Biosystems® by Thermo Fisher Scientific, Foster City, CA, USA), primer 5′-AGATGAGTCTTCTAACCGTCTCTG (Sigma-Aldrich, St. Louis, MO, USA), probe 5′-[6FAM] TCAGGCCCCCTCAAAGCCGA [BHQ1] (Sigma-Aldrich, St. Louis, MO, USA), and 2 μL of sample RNA for a final well volume of 10 μL. Each sample was processed at least three times on a 384 well plate with a minimum of three negative control wells and three positive control wells. We used LPAIV H5N9 stock virus in a 10-fold dilution on each plate in three replicates to create a reference standard curve (Additional File 13). Ct values less than 40 were considered positive for virus. Using QuantStudio™ 6 and 7 Flex Real-Time PCR Software System v1.3, we calculated the standard curve, which was used to estimate virus quantity of each sample by correlating Ct values to 50% egg infectious dose per milliliter (EID_50_/mL). The reported limit of detection is 0.1 EID_50_ [54]; therefore, any samples with undetectable viral RNA were considered negative and assumed to be 0.00 EID_50_/mL. Virus quantity for each sample was averaged across sample replicates. Failed wells and suspected contaminated wells were removed from final calculations.

The quantification limit of the stock virus 10-fold dilution was approximately 400 EID_50_; however, 21% of our samples were detected to have positive virus between this threshold and 0.1 EID_50_. To validate the stability of our statistical analysis, multiple value random imputation [55] was used for any sample with positive virus between 0.1 and 400 EID_50_, and statistical analysis was repeated. Methods and results of this validation technique are outlined in supplemental material (Additional File 9).

### Lectin histochemistry

We used lectin histochemistry to detect SAα2,3Gal in formalin fixed and paraffin embedded tissues of the intestines and bursa of Fabricius of each bird. *Maackia amurensis* I (MAL I) agglutinin is a plant lectin which binds specifically to Siaα2-3Galβ1-4Glc(NAc) [37, 56] and has been used in multiple receptor distribution studies in ducks and other influenza hosts [57, 58] to detect SAα2,3Gal. MAL II, which specifically binds Siaα2-3Galβ1–3 (Neu5Acα2–6) GalNAc [37], is another lectin commonly used in place of, or in conjunction with MAL I [13, 17, 18, 59]. Trial protocols were tested to determine the proper concentration of each lectin needed for proper binding and visual staining of SAα2,3Gal. The trial protocol resulted in a determined concentration for MAL I, but not MAL II; hence MAL I was the only lectin used given that H5 LPAIVs have similar affinity for the receptors targeted by each lectin [36, 37]; furthermore, any lack of specificity for sialic acid receptors is shared by both lectins [37].

Paraffin embedded tissue (duodenum, jejunum, ileum, cecum, colon, and bursa of Fabricius) from each bird was sectioned and stained with biotinylated lectin MAL I (Vector Laboratories, Burlingame, CA, USA), using previous described methods [17, 58] with minor modifications. Paraffin embedded tissue sections were deparaffinized and processed with the EnVision FLEX Target Retrieval Solution, Low pH kit wash buffers, blocking agents, and DAB plus chromogen working solution (Agilent, Dako Omnis, Santa Clara, CA, USA). Tissue sections were first treated with 100 μL of 3% Peroxide Block, then Avidin/Biotin blocking agent (Agilent, Dako Omnis, Santa Clara, CA, USA), and protein blocking. The tissue sections were incubated in 100 μL of MAL I for 32 min, and then treated for 20 min in 100 μL of streptavidin peroxidase (Agilent, Dako Omnis, Santa Clara, CA, USA). The working solution (200 μL) was applied and tissue sections were finally counter stained with 100 μL of hematoxylin (Gill’s III, 1:10 dilution) (Astral Diagnostics Incorporated, West Deptford, New Jersey, USA). All tissue sections stained in the same batch were also stained with a known positive control of duck (*Anas platyrhynchos domesticus*) tissue.

We assessed the abundance of SAα2,3Gal in the proximal intestine (combined duodenum and jejunum), ileum, cecum, colon, and bursa of Fabricius by estimating occurrence frequency of lectin stained cells. We estimated the percentage of lectin stained cells per 5 mm sections of tissue and cell type via an ordinal visual scoring method commonly used in histochemistry [60], which we called “lectin score.” Using brightfield microscopy (400x), we looked specifically at the bursa epithelial cells, and three cell types in each intestinal tissue: the brush border, villi enterocytes, and crypt enterocytes. We scored as many fields of view (FOV) as possible with a maximum of 10 FOVs per cell type in each tissue (Additional Files 14 and 15). Each FOV received a score based on the estimated percentage of cells stained in that FOV. A score of zero indicated that no cells were stained in that field of view. A score of 5 indicated that 1–10% of cells were stained. A score of 35 indicated that 11–60% of cells were stained. A score of 80 indicated that 61–100% of cells were stained. The scores for the FOVs were averaged to obtain a single score for each tissue and cell type, providing 13 separate lectin scores per bird. All samples were scored by the same individual (AD) to eliminate inter-observer error. In some cases, the tissue had become autolyzed and could not be scored, which was more common for the ileum and bursa tissues in mallards possibly due to longer processing times compared to teals.

Since the scoring method used to quantify the frequency of SAα2,3Gal was based off four categories of scores compared to a quantitative continuous scale, we validated our scoring method with the absolute counts of stained cells for 20 randomly selected birds from mallard groups MT1, MT2, and MT5. For each tissue, a single observer (AD) counted the number of stained cells out of 500 cells for each cell type of the ileum and colon, then calculated the percentage. With a total of 108 counts for 20 birds, we found high agreement between our scoring method and the absolute counts (R^2^ = 0.79, *p* < 0.001).

### Statistical analysis

Statistical software R version 3.4.4 [61] was used for all statistical analyses. *P*-values of less than 0.05 were considered statistically significant and assumptions of normality were met by Log_10_(value + 1) transforming all virus titer and lectin histochemistry data. These methods were performed for both mallards and teals unless otherwise indicated. All analyses only included virus titer data collected on one to five DPI, when the majority of virus was shed.

For birds sacrificed during the first five DPI, we used simple linear regression to analyze the relationship between virus titers in the cloacal swab, ileum tissue, and bursa tissue, since all three of these variables were collected at the time the bird was sacrificed. Only the cloacal swab collected on the day the bird was sacrificed was used in this analysis. Six total comparisons were evaluated, three for each species (swab vs. ileum, swab vs. bursa, ileum vs. bursa). In each comparison, the effect of DPI was also evaluated.

A repeated measures, linear mixed effects model [62] was used to test for differences in virus titer or lectin score between species, between sexes, and between control and infected birds (lectin score only). To account for repeated measures of individuals birds, each model was adjusted with a random intercept for each bird. Additionally, when variances of virus titer were different between the factors of the main effects, the model was adjusted to allow for unequal variances. Differences in variance were detected using the Fligner-Killeen test [63]. ANOVA tables were visualized, and the post-hoc Tukey’s test was used to assess pairwise differences.

To analyze the effect of species on virus titer, species, DPI, and the species*DPI interaction were included in the model. To analyze the effect of sex on virus titer, we assembled two separate models: one for mallards and one for teals. Sex and DPI, plus their interaction, were included in each model.

To analyze the effect of lectin score on infection status (infected vs. control), mallards and teals were assessed in two separate models. For each species, infection status and tissue/cell-type, plus their interaction, were included in their respective model.

Using data from infected birds only, we also assessed species and sex-based differences in lectin score. To analyze the effect of species on lectin score, species, tissue/cell type, and their interaction, were included in the model. To analyze the effect of sex on lectin score, mallards and teals were analyzed in separate models. Sex and tissue/cell type, plus their interaction, were included in each model.

We also looked at lectin score correlations between cell types within intestinal tissue type using Pearson’s r coefficient. We considered cell types within a tissue type (proximal, ileum, cecum, colon) with a coefficient of 0.8 or higher to indicate a strong correlation. If all three cell types within a tissue were highly correlated, we used PCA to reduce the data into one component variable we called “[tissue type] PC.” Each PC variable accounted for greater than 80% of the variation between the cell types of that particular tissue. PC variables generated from the PCA were used in the MLR models to determine the relationship between virus titer and lectin score.

Virus titer and lectin score relationship was determined by assessing three different MLR models for each species using virus titer as the dependent variable. The virus titer variable in the first model consisted of virus titers from cloacal swabs collected on the DPI each bird was sacrificed. The second model used virus titers in ileum tissue, and the third model used virus titers in bursa tissue. Independent variables for the cloacal swab virus titer model consisted of the lectin score variables, the principal components described above (when appropriate), and five control variables: sex, BCS, LPAIV treatment group, body mass in grams at 55 days after hatch, and inoculation age in days. Independent variables for the ileum virus titer model included only the ileum lectin score variables and the five control variables. Only the bursa epithelium lectin score variable and the five control variables were included in the bursa virus titer model.

To determine the best fitting MLR model for each dependent variable, we followed a consistent procedure. Global linear models were tested for each dependent variable separately. To select parsimonious model fits to the data, we used stepwise variable selection based on the generalized Akaike’s Information Criterion (AIC). We then used variance inflation factor (VIF) scores to identify problematic co-linear predictors from the stepwise-chosen models. Independent variables with VIFs > 3.0 were determined problematic and were removed from the model one at a time until all VIFs < 3.0 [64]. When two VIFs were > 3.0 and < 1.0 in difference, we tested alternative models. Stepwise variable selection was used for each model to ensure the best fitting model. Residual plots were reviewed. For each of the three dependent variables, the model with both the lowest AIC, highest adjusted R^2^, and satisfactory residual patterns (e.g., no linear or nonlinear trend in residuals, little to no heterogeneous variance in residuals, and no suspected outlier observations) was chosen as the best fitting model to the data.

## Supplementary Information

**Additional file 1.** All analyzed data for LPAIV H5N9 infected and control blue-winged teals and mallards. NA indicates missing data.

**Additional file 2 **Mean virus titer + 95% confidence interval for (a) species, (b) days post infection (DPI), and (c) the interaction of species and DPI for mallard and blue-winged teal cloacal swab samples one to five DPI.

**Additional file 3 **Mean virus titer + 95% confidence intervals for (a,c) sex, and (b,d) the interaction of sex and days post infection (DPI) for male (M) and female (F) mallard and teal blue-winged teal cloacal swab samples one to five DPI.

**Additional file 4 **Mean lectin scores + 95% confidence intervals for intestinal tissues proximal (duodenum and jejunum), ileum, cecum, and colon for LPAIV H5N9 infected male (M) and female (F) mallards and blue-winged teals.

**Additional file 5.** Mallard PCA results for proximal and cecum lectin scores.

**Additional file 6.** Residual Plots and AIC table for mallard cloaca swab virus titer multiple linear regression model.

**Additional file 7.** Residual plots and AIC table for mallard ileum virus titer multiple linear regression model.

**Additional file 8.** Residual plots and AIC table for blue-winged teal cloaca swab virus titer multiple linear regression model.

**Additional file 9.** Quantitative limit validation methods and results.

**Additional file 10.** Residual plots and AIC table for blue-winged teal ileum virus titer multiple linear regression model.

**Additional file 11.** Residual plots and AIC table for blue-winged teal bursa virus titer multiple linear regression model.

**Additional file 12.** Pearson’s r correlation matrix for mallard lectin histochemistry scores.

**Additional file 13.** RTPCR raw data for virus titer quantities.

**Additional file 14.** Lectin score raw data for mallards.

**Additional file 15.** Lectin score raw data for blue-winged teals.

## Data Availability

All data analyzed during this study are included in this published article (Additional Files 1, 13, 14, and 15).

## Abbreviations

AIV: Avian influenza virus
HPAIV: Highly pathogenic avian influenza virus
LPAIV: Low pathogenic avian influenza virus
SAα2,3Gal: alpha-2,3 sialic acid receptor
MAL I: *Maackia amurensis* I
MAL II: *Maackia amurensis* II
DPI: Days post infection
Est.: Slope parameter estimate
PCA: Principle component analysis
MLR: Multiple linear regression
BCS: Body condition score
MSU: Michigan State University
ECE: Embryonated chicken eggs
EID50: 50% egg infection dose
DMEM: Dulbecco’s modified eagle medium
FOV: Fields of view
AIC: Akaike’s information criteria
VIF: Variance inflation factor
