# Supplementary figures and images for: The association between SAα2,3Gal occurrence frequency and avian influenza viral load in mallards (Anas platyrhynchos) and blue-winged teals (Spatula discors)
Source: BMC Vet Res. 2020 Nov 10;16:430. doi: 10.1186/s12917-020-02642-7 (PMC7653716; doi:10.1186/s12917-020-02642-7)

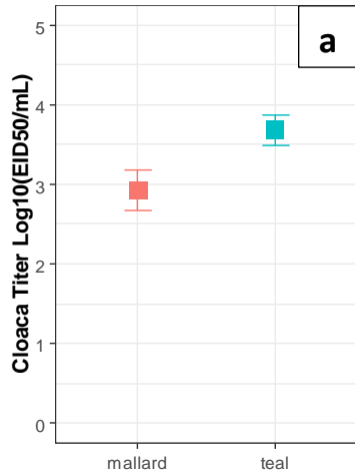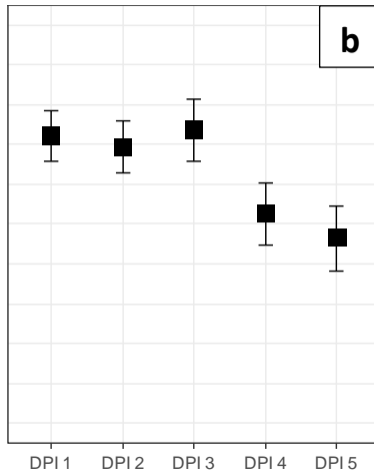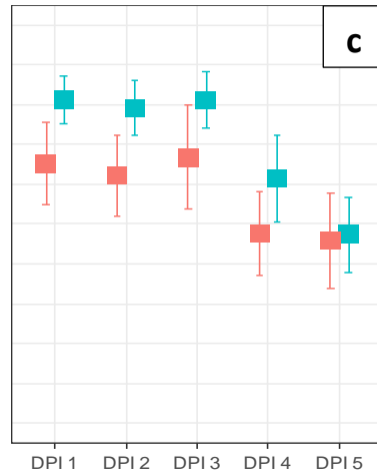

Species

mallard

teal

Supplement: Supplementary file 2 — Additional file 2 Mean virus titer + 95% confidence interval for (a) species, (b) days post infection (DPI), and (c) the interaction of species and DPI for mallard and blue-winged teal cloacal swab samples one to five DPI. [file 12917_2020_2642_MOESM2_ESM.pdf]

## Mallard

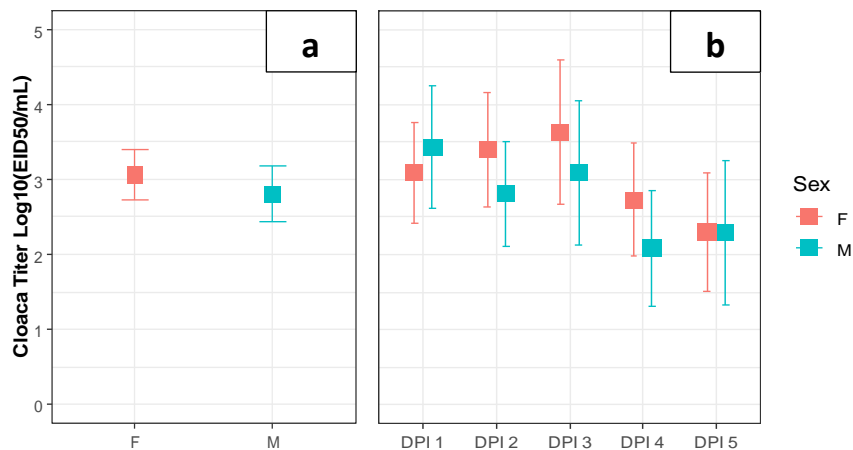

## Teal

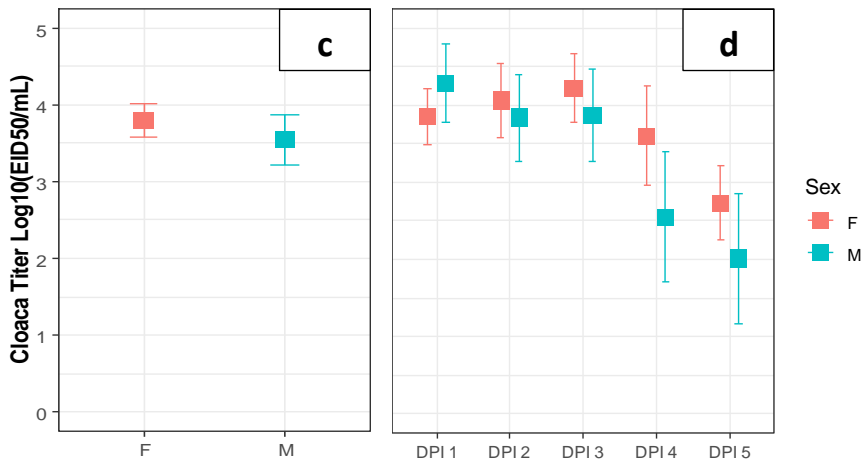

Supplement: Supplementary file 3 — Additional file 3 Mean virus titer + 95% confidence intervals for (a,c) sex, and (b,d) the interaction of sex and days post infection (DPI) for male (M) and female (F) mallard and teal blue-winged teal cloacal swab samples one to five DPI. [file 12917_2020_2642_MOESM3_ESM.pdf]

Mallard

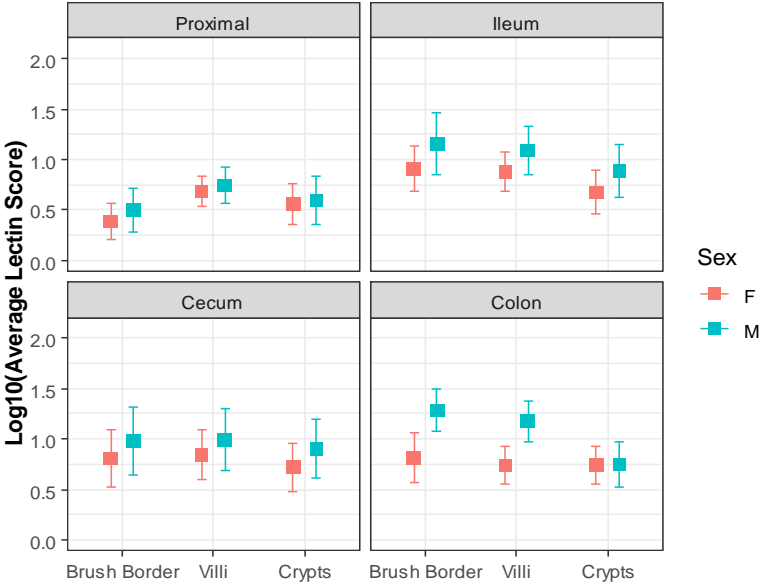

Teal

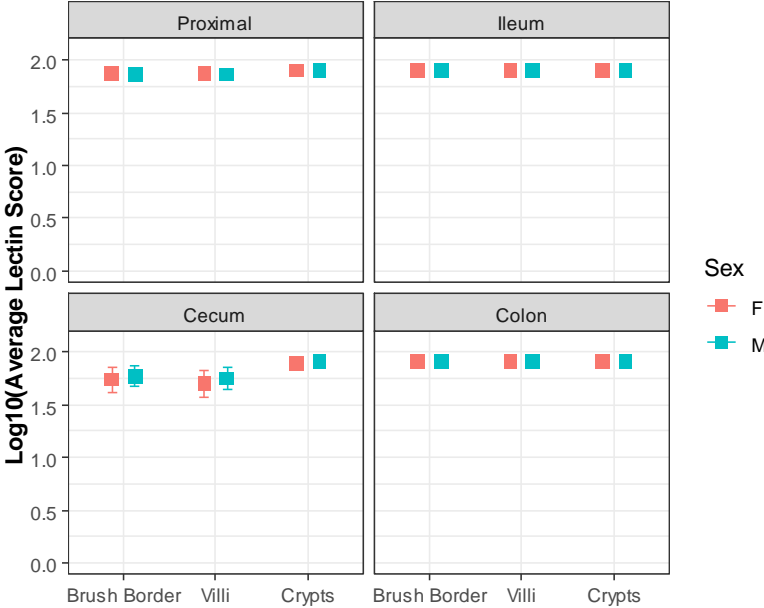

Supplement: Supplementary file 4 — Additional file 4 Mean lectin scores + 95% confidence intervals for intestinal tissues proximal (duodenum and jejunum), ileum, cecum, and colon for LPAIV H5N9 infected male (M) and female (F) mallards and blue-winged teals. [file 12917_2020_2642_MOESM4_ESM.pdf]
