# Supplementary material for: The association between SAα2,3Gal occurrence frequency and avian influenza viral load in mallards (Anas platyrhynchos) and blue-winged teals (Spatula discors)
Source: BMC Vet Res. 2020 Nov 10;16:430. doi: 10.1186/s12917-020-02642-7 (PMC7653716; doi:10.1186/s12917-020-02642-7)

Mallard - Cloaca Swab Virus Titer MLR model supporting material

Global Model: $Swab\ Titer = 1 + factor(Sex) + mass + age + BCS + factor(group) + ProximalPC + Ileum\ Villi + Ileum\ Brush\ Border + Ileum\ Crypts + CecumPC + Colon\ Villi + Colon\ Brush\ Border + Colon\ Crypts$

Final Model: $Swab\ Titer = 1 + factor(Sex) + ProximalPC + Ileum\ Villi + Ileum\ Brush\ Border$

Step	Model	AIC	ΔAIC
NA	Global	26.83	NA
1	Global – group	23.12	3.70
2	Step 1 – Ileum Crypts	21.14	1.99
3	Step 2 – Cecum PC	19.20	1.94
4	Step 3 – age	17.83	1.37
5	Step 4 – mass	16.11	1.72
6	Step 5 – Colon Crypts	14.55	1.56
7	Step 6 – BCS	14.07	0.48
8	Step 7 – Colon BB	13.22	0.86
9	Step 8 – Colon Villi	11.44	1.77

Residual Plots

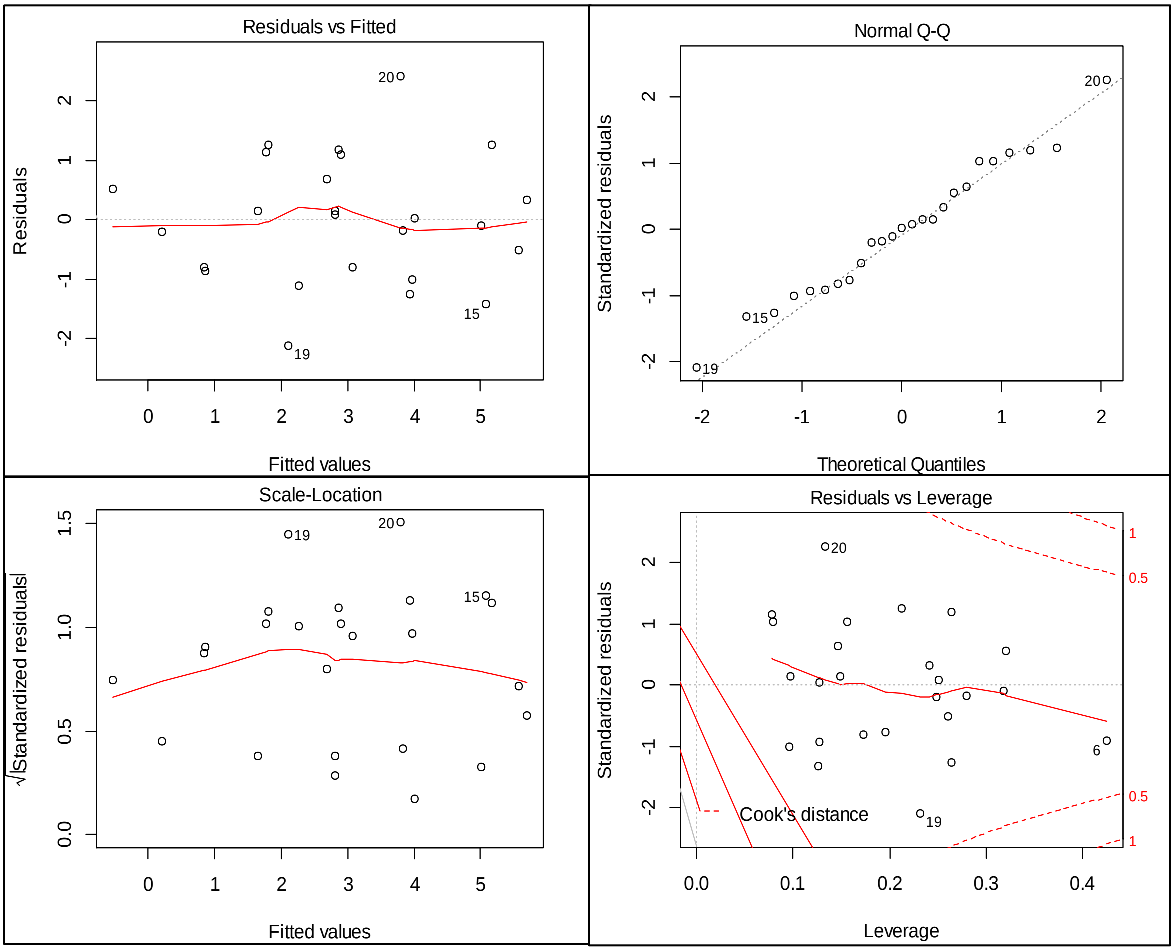

Supplement: Supplementary file 6 — Additional file 6. Residual Plots and AIC table for mallard cloaca swab virus titer multiple linear regression model. [file 12917_2020_2642_MOESM6_ESM.pdf]
