# Supplementary material for: The association between SAα2,3Gal occurrence frequency and avian influenza viral load in mallards (Anas platyrhynchos) and blue-winged teals (Spatula discors)
Source: BMC Vet Res. 2020 Nov 10;16:430. doi: 10.1186/s12917-020-02642-7 (PMC7653716; doi:10.1186/s12917-020-02642-7)

Mallard - Ileum Virus Titer MLR model supporting material

Global Model: $Ileum\ Titer = 1 + factor(Sex) + mass + age + BCS + factor(group) + Ileum\ Villi + Ileum\ Brush\ Border + Ileum\ Crypts$

Final Model: $Ileum\ Titer = 1 + factor(Sex) + ProximalPC + Ileum\ Villi + Ileum\ Brush\ Border$

Step	Model	AIC	ΔAIC
NA	Global	44.13	NA
1	Global - group	40.65	3.48
2	Step 1 - age	38.65	2.00
3	Step 2 - BCS	36.69	1.96
4	Step 3 – Ileum Crypts	34.90	1.79
5	Step 4 - mass	33.57	1.32

Residual Plots

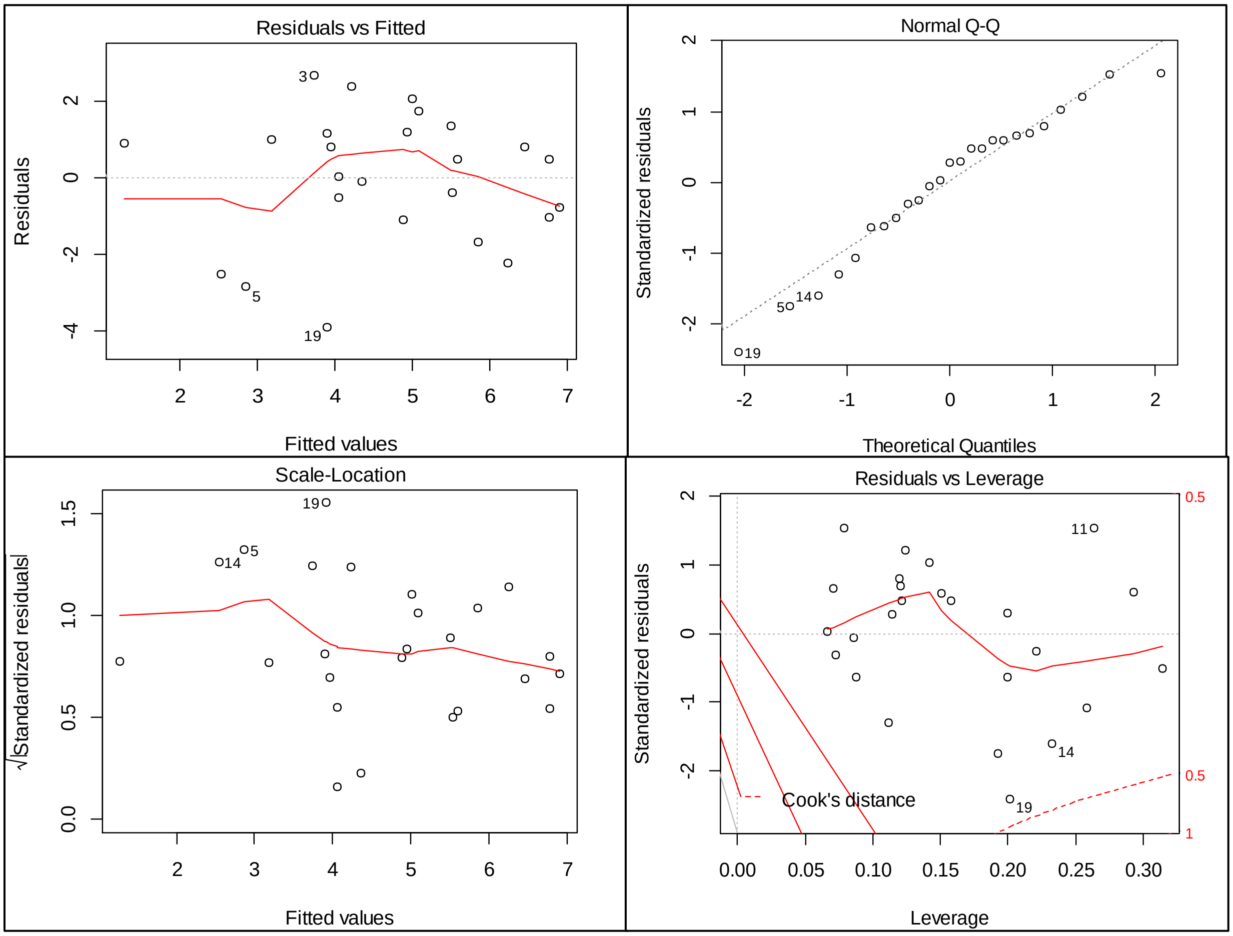

Supplement: Supplementary file 7 — Additional file 7. Residual plots and AIC table for mallard ileum virus titer multiple linear regression model. [file 12917_2020_2642_MOESM7_ESM.pdf]
