# Supplementary material for: The association between SAα2,3Gal occurrence frequency and avian influenza viral load in mallards (Anas platyrhynchos) and blue-winged teals (Spatula discors)
Source: BMC Vet Res. 2020 Nov 10;16:430. doi: 10.1186/s12917-020-02642-7 (PMC7653716; doi:10.1186/s12917-020-02642-7)

Blue-winged Teal - Cloaca Swab Virus Titer MLR model supporting material

Global Model: $Swab\ Titer = 1 + \ factor(Sex) + mass\ + age + BCS + factor(group) + Proximal\ Villi + Proximal\ Brush\ Border + Proximal\ Crypts\ + Ileum\ Villi + Cecum\ Villi + \ Cecum\ Brush\ Border + Cecum\ Crypts + Colon\ Villi + Colon\ Brush\ Border + Colon\ Crypts + Bursa$

Final Model: $Swab\ Titer = 1 + factor(Sex) + mass + BCS + factor(group) + Proximal\ Crypts + Bursa$

Y	N	R^2	X	EST. (95% CI) LOG10(EID50/ML)	P
TEAL CLOACA SWAB VIRUS TITER	32	0.61	Intercept	8.35 (-7.50 to 24.20)	0.288
			Sex (Male)	-0.60 (--1.30 to 0.09)	0.086
			mass	0.01 (0.01 to 0.02)	0.001
			BCS	-0.81 (-1.50 to -0.12)	0.023
			group (T3)	-0.12 (-0.97 to 0.73)	0.780
			group (T5)	-1.92 (-2.67 to -1.17)	<0.001
			Proximal Crypts	-4.88 (-12.23 to 2.54)	0.189
			Bursa	1.66 (-0.11 to 3.42)	0.064

Y = dependent variable, N = number of individual birds in model, X = independent variables in final model, CI = 95% confidence interval, p = p-value. Proximal includes the duodenum and jejunum. BCS = Body Condition Score. Group and Sex were treated as factors in each model, and if present in final model, group T1 and females are represented in the intercept.

Step	Model	AIC	ΔAIC
NA	Global	-0.09	NA
1	Global – Colon Crypts	-0.09	0.00
2	Step 1 – Colon Villi	-0.09	0.00
3	Step 2 – Colon BB	-0.09	0.00
4	Step 3 – Ileum Villi	-0.09	0.00
5	Step 4 – Proximal BB	-2.09	2.00
6	Step 5 – Cecum Villi	-4.04	1.95
7	Step 6 – Cecum Crypts	-5.73	1.69
8	Step 7 - age	-7.46	1.73
9	Step 8 – Cecum BB	-8.21	0.75
10	Step 9 – Proximal Villi	-8.31	0.10

Residual Plots

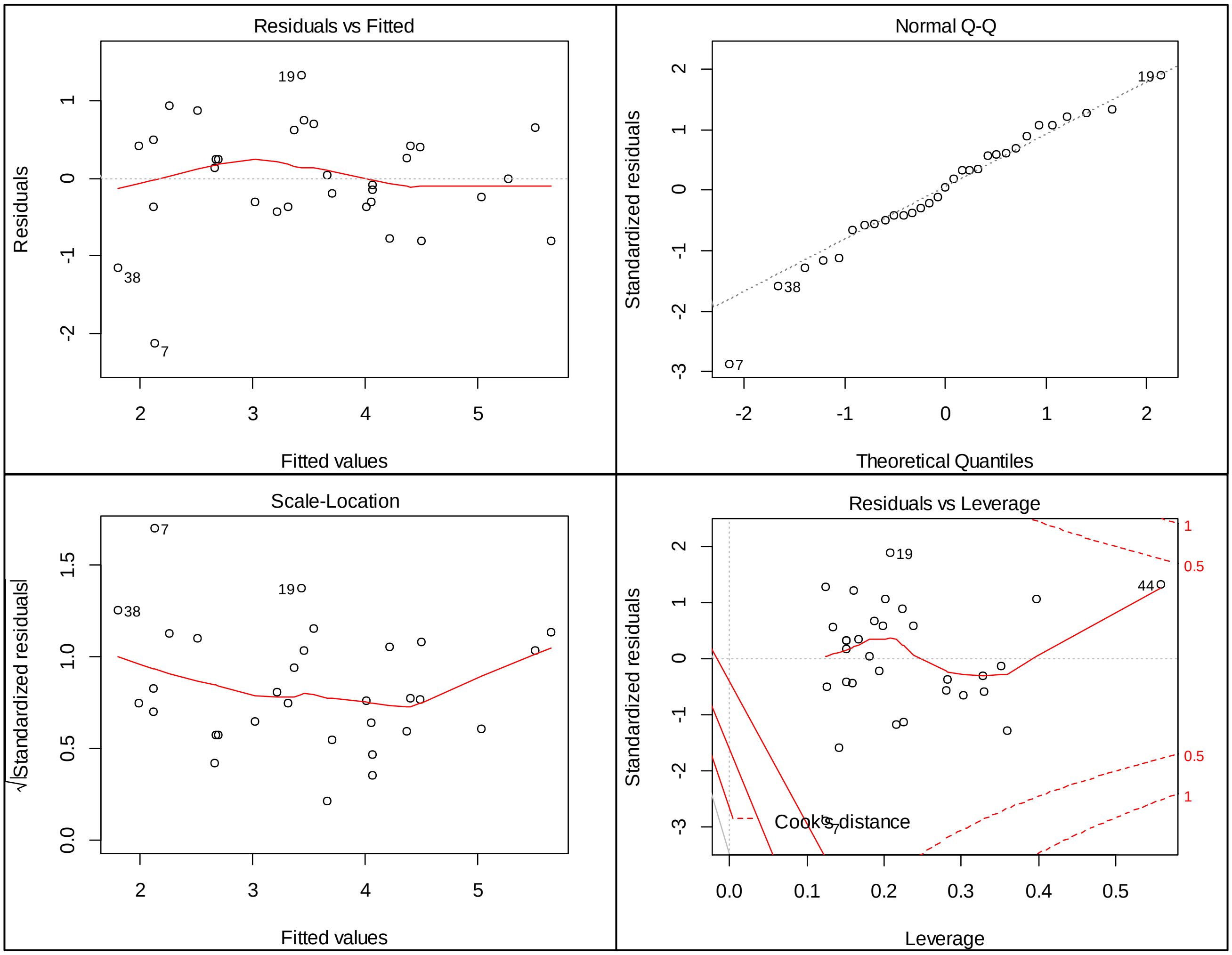

Supplement: Supplementary file 8 — Additional file 8. Residual plots and AIC table for blue-winged teal cloaca swab virus titer multiple linear regression model. [file 12917_2020_2642_MOESM8_ESM.pdf]
