# Supplementary material for: The association between SAα2,3Gal occurrence frequency and avian influenza viral load in mallards (Anas platyrhynchos) and blue-winged teals (Spatula discors)
Source: BMC Vet Res. 2020 Nov 10;16:430. doi: 10.1186/s12917-020-02642-7 (PMC7653716; doi:10.1186/s12917-020-02642-7)
Supplement: Supplementary file 9 — Additional file 9. Quantitative limit validation methods and results. [file 12917_2020_2642_MOESM9_ESM.docx]

**RT-PCR virus titer quantification limit validation**

**Methods**

To evaluate the effect of using estimated virus quantities, (values >DL of 0.10 EID_50_ and <QL of 400 EID_50_) in statistical modeling on parameter estimates, using excel, any qPCR value for virus titer between 0.1 and 400 EID_50_ was replaced with a random number using =RANDBETWEEN(0.1, 400). All other virus titer values remained the same. Similar to the methods outlined in the main manuscript text, all values were transformed using Log10(value +1). Statistical analyses involving virus titer data were repeated using these new data with the substituted random numbers.

**Results of Validation Method**

*Relationship of Virus Titers in Cloacal Swabs, Ileum, and Bursa*

Cloaca swab virus titer, ileum virus titer, and bursa virus titer were all significantly positively (p < 0.001) related to each other for both mallard and teal. In mallards, statistically significant positive relationships were observed between ileum virus titer and cloacal swab virus titer for all treatment groups (MT1, R^2^ = 0.40, p = 0.007; MT2, R^2^ = 0.66, p = 0.003; and MT5, R^2^ = 0.58, p < 0.001). Statistically significant positive relationships were observed between bursa virus titer and cloacal swab virus titer for treatment groups MT1 (R^2^ = 0.39, p = 0.007) and MT5 (R^2^ = 0.60, p < 0.001). Only MT5 (R^2^ = 0.71, p < 0.001) had a statistically significant positive relationship between ileum virus titer and bursa virus titer. In teals, the only statistically significant positive relationship for treatment groups was observed for BT1 (R^2^ = 0.35, p = 0.032) between cloacal swab virus titer and bursa virus titer. These results are to be compared with the results from the main text of the manuscript, where the results are similar.

*Species and Sex-based differences in Viral Shedding*

Statistically significant differences in viral shedding were found between mallards and teals, but not between males and females within species. Mallards had higher individual variation (larger 95% confidence intervals) observed in cloacal swab viral titers than teals (Table 1, Figure 2). For both species, cloacal swab virus titers on one, two, and three DPI were significantly higher statistically than virus titers on four and five DPI (F_4,242_ = 16.07, p < 0.001). Teals shed significantly more virus than mallards (F_1,102_ = 11.79, p = 0.001) with no interaction between species and DPI (F_4,242_ = 0.70, p = 0.592). No sex-based differences were observed in cloacal swab virus titers for either species (mallard: F_1,58_ = 0.03, p = 0.865; teal: F_1,42_ = 0.67, p = 0.419) with no the interaction between sex and DPI (mallard: F_4,138_ = 0.13, p = 0.970; teal: F_4,96_ = 2.24, p = 0.070; Figure S2). These results are to be compared with the results from the main text of the manuscript, where the results are similar.

*Relationship between Lectin Histochemistry Score and Virus Titer – Mallard*

For cloacal swab virus titer, initial stepwise variable selection rendered a model which included sex, ileum villi, and ileum brush border (AIC = 10.37, ΔAIC = 0.08). This reduced model was tested for co-linearity issues and residual plots were evaluated with no statistically serious problems detected, so the reduced model was selected as the best fitting model (R^2^ = 0.61, p < 0.001).

For ileum virus titer, initial stepwise variable selection rendered a model which included sex, ileum villi, and ileum brush border (AIC = 33.57, ΔAIC = 1.33). This reduced model was tested for co-linearity issues and residual plots were evaluated with no statistically serious problems detected, thus this model (R^2^ = 0.33, p < 0.010) was selected as the best fitting model.

These results are to be compared with the results from the main text of the manuscript, where the results are similar. See ANOVA table comparisons below.

*Relationship between Lectin Histochemistry Score and Virus Titer – Teal*

For cloacal swab virus titer, initial stepwise variable selection rendered a model which included mass, BCS, group, proximal villi, proximal crypts, cecum villi, and bursa (AIC = -16.97, ΔAIC = 0.71). This reduced model was tested for co-linearity issues and residual plots were evaluated. The model did not meet the assumption of normality; therefore, the insignificant terms, cecum villi and bursa were removed from the model. The final model, which included mass, BCS, group, proximal villi, and proximal crypts (R^2^ = 0.61, p < 0.001) was selected as the best fitting model. These results are to be compared with the results from the main text of the manuscript, where the results are slightly different. See ANOVA table comparisons below.

For teal ileum virus titer and virus titer, no samples fell below the limit of quantification; therefore, the quantification limit validation was not performed.

**Conclusions**

The quantification limit validation method was used to check the stability of our statistical analyses and MLR models, given our qPCR results for our stock virus standard curve 10-fold dilution quantification limit was approximately 400 EID_50_. The results from the validation method confirms the stability of statistical analyses for the species and sex-based differences as well as the stability of the MLR models for mallard cloaca swab virus titer and mallard ileum virus titer. However, we did receive different results when the validation method was tested for teal cloaca swab virus titer, where a final model with slightly different parameters was observed. We conclude that while the results of the mallard MLR models are stable, we cannot confirm the results for the teal cloaca swab virus titer model to be stable. We suggest this is due to the overall lack of variation in teal lectin scores, where most individuals scored the maximum possible lectin score for all tissues/cell types, and only a few individuals had lower scores.

**ANOVA Table Comparisons**

|  | Reported Data (extrapolated values below QL) | QL Validation Data (random values assigned when result <QL) |
| --- | --- | --- |
| Mallard  Cloaca  Swab  Virus  Titer | Estimate CI P  Intercept 1.37 0.14-2.60 0.031  (Sex)M 1.66 0.60t-2.73 0.004  PC1.Proximal 0.50 -0.22-1.22 0.166  Ileum.Villi 2.93 1.142-4.44 0.001  Ileum.BB* -1.96 -3.12-(-0.80) 0.002 | Estimate CI P  Intercept 1.45 0.41-2.50 0.009  (Sex)M 1.73 0.75-2.71 0.001  Ileum.Villi 3.21 1.89-4.52 <0.001  Ileum.BB -2.19 -3.32-(-1.06) 0.001 |
| Mallard  Ileum  Virus  Titer | Estimate CI P  Intercept 2.86 1.20-4.52 0.001  (Sex)M 1.36 -0.19-2.91 0.083  Ileum.Villi 3.27 1.18-5.36 0.004  Ileum.BB -1.93 -3.73-(-0.14) 0.036 | Estimate CI P  Intercept 2.86 1.20-4.52 0.002  (Sex)M 1.36 -0.19-2.91 0.083  Ileum.Villi 3.27 1.18-5.36 0.004  Ileum.BB -1.93 -3.73-(-0.14) 0.036 |
| Teal  Cloaca  Swab  Virus  Titer | Estimate CI P  Intercept 8.35 -7.50-24.20 0.288  (Sex)M -0.60 -1.30-0.09 0.086  mass 0.01 0.01-0.2 0.001  BCS -0.81 -1.50-(-0.12) 0.023  (group)T3 -0.12 -0.97-0.73 0.780  (group)T5 -1.92 -2.67-(-1.17) <0.001  Prox.Crypts -4.88 -12.23-2.54 0.188  Bursa.LS 1.66 -0.11-(-3.42) 0.064  CI = 95% Confidence Interval, BB = brush border, | Estimate Std. Error t value Pr(>\|t\|)  Intercept 18.14 5.51-30.77 0.007  mass 0.01 0.01-0.02 0.001  BCS -0.92 -1.55-(-0.29) 0.006  (group)T3 -0.66 -1.44-0.13 0.099  (group)T5 -179 -2.47-(-1.10) <0.001  Prox.Villi 3.55 -0.15-7.25 0.059  Prox.Crypts -11.54 -19.39-(-3.68) 0.006 |

**Residual plots for validation method data**

| Mallard cloaca swab virus titer – QL validation |
| --- |

| Mallard ileum virus titer – QL validation |
| --- |

| Blue-winged Teal cloaca swab virus titer – QL Validation |
| --- |
