# Supplementary material for: The association between SAα2,3Gal occurrence frequency and avian influenza viral load in mallards (Anas platyrhynchos) and blue-winged teals (Spatula discors)
Source: BMC Vet Res. 2020 Nov 10;16:430. doi: 10.1186/s12917-020-02642-7 (PMC7653716; doi:10.1186/s12917-020-02642-7)

Blue-winged teal Ileum Virus Titer MLR model supporting material

Global Model: $Ileum\ Titer\ = 1 + factor(Sex) + mass\ + age + BCS + factor(group) +\ Ileum\ Villi$

Final Model: $Ileum\ Titer\ = 1 +\ BCS + factor(group)$

STEP	MODEL	AIC	ΔAIC
NA	Global	38.3996	NA
1	Global – Ileum Villi	38.3996	0
2	Step 1 - Sex	36.39985	1.999755
3	Step 2 - mass	34.40121	1.998643
4	Step 3 - age	32.41009	1.991114

Y	N	R^2	X	EST. (95% CI) LOG10(EID50/ML)	P
TEAL ILEUM TITER	32	0.44	Intercept	10.03 (5.71 to 14.35)	<0.001
			BCS	-0.86 (-1.91 to 0.19)	0.103
			group (T3)	-1.05 (-2.54 to 0.47)	0.169
			Group (T5)	-3.42 (-4.88 to -1.97)	<0.001

Y = dependent variable, N = number of individual birds in model, X = independent variables in final model, CI = 95% confidence interval, p = p-value. BCS = Body Condition Score. Group was treated as a factor and group T1 is represented in the intercept.

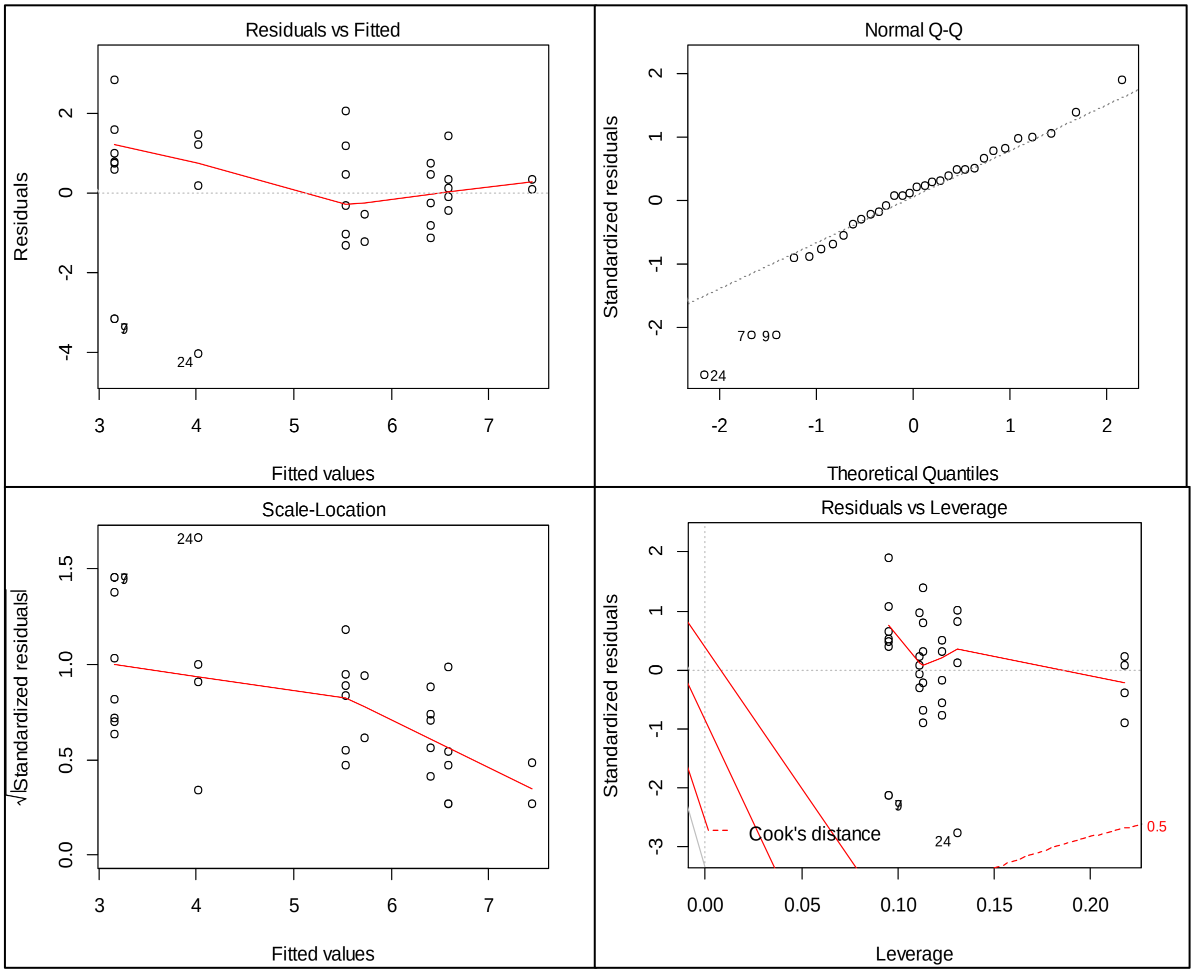

Supplement: Supplementary file 10 — Additional file 10. Residual plots and AIC table for blue-winged teal ileum virus titer multiple linear regression model. [file 12917_2020_2642_MOESM10_ESM.pdf]
