# Supplementary material for: The association between SAα2,3Gal occurrence frequency and avian influenza viral load in mallards (Anas platyrhynchos) and blue-winged teals (Spatula discors)
Source: BMC Vet Res. 2020 Nov 10;16:430. doi: 10.1186/s12917-020-02642-7 (PMC7653716; doi:10.1186/s12917-020-02642-7)

Blue-winged teal Bursa Virus Titer MLR model supporting material

Global Model: $Bursa\ Titer = 1 + factor(Sex) + mass + age + BCS + factor(group) + Bursa$

Initial Model: $Bursa\ Titer = 1 + BCS + mass + factor(group)$

Residual Plots for initial multiple linear regression model *Blue-winged teal Bursa Titer.*

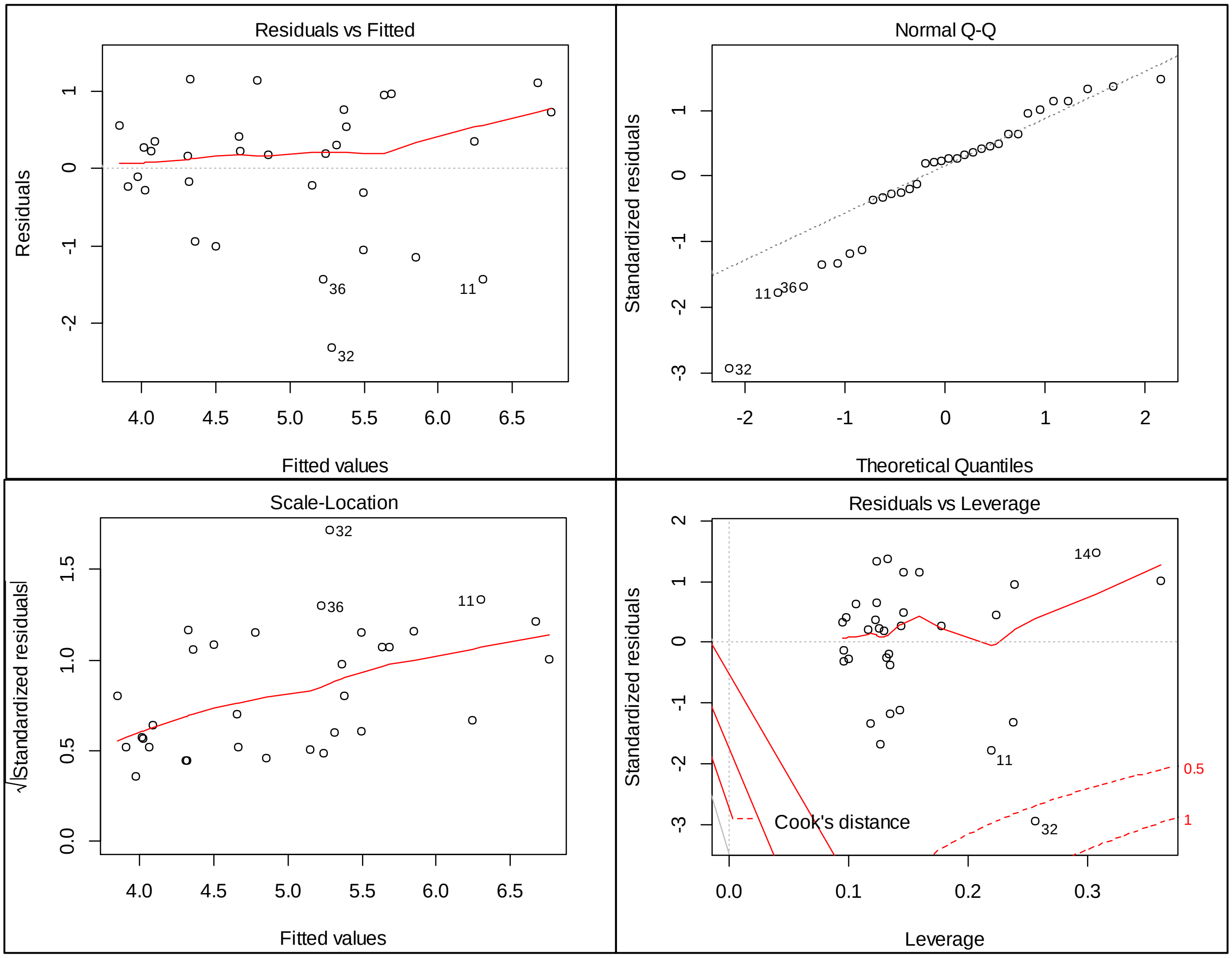

Final Model: $Bursa\ Titer = 1 + BCS + factor(group)$

STEP	MODEL	AIC	ΔAIC
NA	Global	2.14	NA
1	Global - Sex	0.15	2.00
2	Step 1 - Bursa	-1.60	1.75
MANUAL	Step 2 - mass	-0.25	-1.35

Y	N	R^2	X	EST. (95% CI) LOG10(EID50/ML)	P
TEAL BURSA TITER	32	0.37	Intercept	7.52 (4.92 to 10.11)	< 0.001
			BCS	-0.38 (-1.01 to 0.24)	0.220
			group (T3)	-1.04 (-1.95 to -0.13)	0.027
			group (T5)	-1.96 (-2.83 to -1.08)	<0.001

Y = dependent variable, N = number of individual birds in model, X = independent variables in final model, CI = 95% confidence interval, p = p-value. BCS = Body Condition Score. Group was treated as a factor and group T1 is represented in the intercept.

Residual Plots for <u>final</u> multiple linear regression model *Blue-winged teal Bursa Titer.*

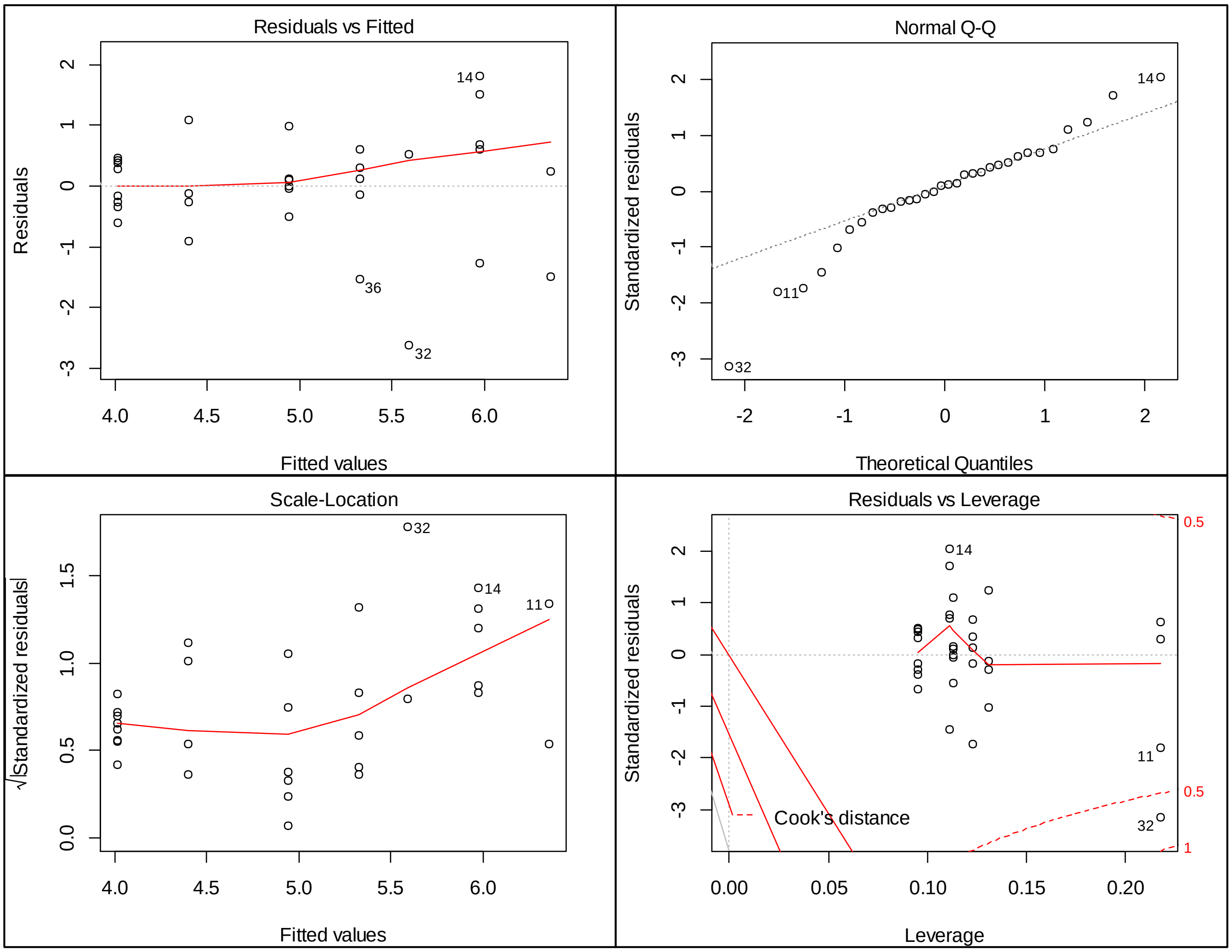

Supplement: Supplementary file 11 — Additional file 11. Residual plots and AIC table for blue-winged teal bursa virus titer multiple linear regression model. [file 12917_2020_2642_MOESM11_ESM.pdf]
